# Dynamic alterations in protein, sensory, chemical, and oxidative properties occurring in meat during thermal and non-thermal processing techniques: A comprehensive review

**DOI:** 10.3389/fnut.2022.1057457

**Published:** 2023-01-11

**Authors:** Waseem Khalid, Aristide Maggiolino, Jasmeet Kour, Muhammad Sajid Arshad, Noman Aslam, Muhammad Faizan Afzal, Parkash Meghwar, Khair-ul-Wajeeha Zafar, Pasquale De Palo, Sameh A. Korma

**Affiliations:** ^1^Department of Food Science, Faculty of Life Sciences, Government College University, Faisalabad, Pakistan; ^2^Department of Veterinary Medicine, University of Bari Aldo Moro, Bari, Italy; ^3^Department of Food Science and Technology, Padma Shri Padma Sachdev Government PG College for Women, Jammu, Jammu and Kashmir, India; ^4^Department of Food Science and Technology, University of Karachi, Karachi, Pakistan; ^5^Department of Chemistry, Government College University, Faisalabad, Pakistan; ^6^Department of Food Science, Faculty of Agriculture, Zagazig University, Zagazig, Egypt; ^7^School of Food Science and Engineering, South China University of Technology, Guangzhou, China

**Keywords:** meat, thermal processing, non-thermal processing, protein changes, sensory changes

## Abstract

Meat processing represents an inevitable part of meat and meat products preparation for human consumption. Both thermal and non-thermal processing techniques, both commercial and domestic, are able to induce chemical and muscle’s proteins modification which can have implication on oxidative and sensory meat characteristics. Consumers’ necessity for minimally processed foods has paved a successful way to unprecedented exploration into various novel non-thermal food processing techniques. Processing of meat can have serious implications on its nutritional profile and digestibility of meat proteins in the digestive system. A plethora of food processing techniques can potentially induce alterations in the protein structure, palatability, bioavailability and digestibility *via* various phenomena predominantly denaturation and Maillard reaction. Apart from these, sensory attributes such as color, crispness, hardness, and total acceptance get adversely affected during various thermal treatments in meat. A major incentive in the adoption of non-thermal food processing is its energy efficiency. Considering this, several non-thermal processing techniques have been developed for evading the effects of conventional thermal treatments on food materials with respect to Maillard reactions, color changes, and off-flavor development. Few significant non-thermal processing techniques, such as microwave heating, comminution, and enzyme addition can positively affect protein digestibility as well as enhance the value of the final product. Furthermore, ultrasound, irradiation, high-pressure processing, and pulsed electric fields are other pivotal non-thermal food processing technologies in meat and meat-related products. The present review examines how different thermal and non-thermal processing techniques, such as sous-vide, microwave, stewing, roasting, boiling, frying, grilling, and steam cooking, affect meat proteins, chemical composition, oxidation, and sensory profile.

## 1. Introduction

Meat has an exceptionally rich nutritional value and serves as an excellent source of several essential amino acids and various micronutrients such as vitamins B_3_ and B_12_, iron, and zinc (1). Meat has been classified into three main categories: red meat, poultry, and seafood. Entire livestock is constituted under red meat including lamb, beef, pork, and goat. Poultry usually refers to white meat that constitutes chicken while fish, crustaceans (viz. crab, lobster) and mollusks (viz. clams, oysters, scallops, mussels) comprise of seafood (2). Muscle, and consequently meat chemical composition, is characterized by a great water content (∼75%) followed by protein (∼20%), fat (∼3%), and soluble non-protein substances (∼2%), with differences among different species considered (3–11). The three major groups of muscular proteins can be myofibrillar, sarcoplasmic, and connective tissue proteins. Myofibrillar proteins constitute 50–55% of total protein whereas 30–34% of the total muscular proteins are represented by sarcoplasmic proteins (12). Oleic acid is the major fatty acid present in meat, is highly predominant in neutral lipids and is prepared from stearic acid with the help of a lipogenic enzyme (i.e., stearoyl Co-A desaturase). The quality of meat products is known to be affected by their composition together with the cooking time and temperature (13). Meat is a heterogenous food group with its composition varying with the meat category. Red meats such as in beef, lamb, pork, and processed meat like burgers and sausages have a higher content of fat compared to chicken. Generally, red meats such as beef and dark-colored meat derived from chicken and turkey are a better source of iron than white meats.

Distinctive heat transmission media has been used for the cooking of meat which includes thermal and non-thermal cooking methods. Thermal treatment involves the application of heat first of all because considered the most effective way of eliminating microorganisms causing food-borne diseases (14), although often not in an adequate manner (15). However, humans have been cooking meat for hundreds of thousands of years to improve its digestibility, to modify the physicochemical profile of meat as well as to prepare foods by using varying levels of temperature that in turn depends upon the product being prepared (16). The most widely used methods are grilling, boiling, pan-frying, roasting, and deep-frying (17). The thermal processing led to an alteration in the structural conformation of protein apart from altering sensorial traits like appearance, flavor, texture, and chemical characteristics of the ingredients, affecting tenderization and toughening (18–20). Heterocyclic aromatic amines, which are known to be potentially rich mutagenic/carcinogenic agents, are formed during meat’s thermal processing and incomplete combustion of constituent organic material (21). Goluch et al. (22) thoroughly investigated the effect of various types of thermal processing techniques viz a viz pan frying, water bath cooking, grilling, and oven convection roasting on the nutritional composition and energy of meat of goose breast. Out of all these techniques, oven convection roasting stood out in terms of lowest energy value and retention of the highest nutritional content with respect to fat and essential minerals such as phosphorus and sodium.

**FIGURE 1:**
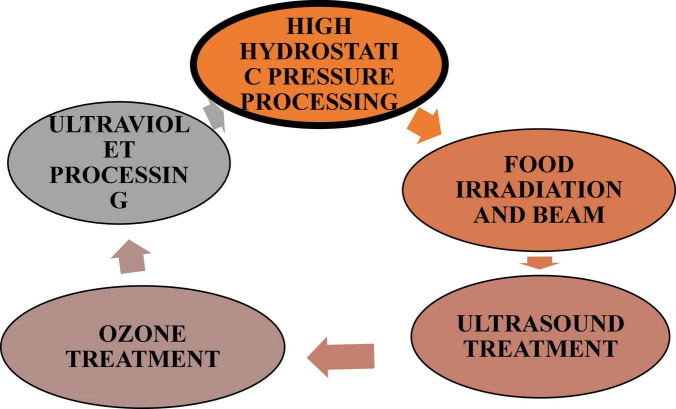
Different types of novel techniques used in meat processing.

The novel non-thermal technology has certain advantages over conventional technologies including enhancing the product quality and safety while also having an advanced level of automation besides having more accurate control over the processing method (23), but the influence of non-thermal technology on the sensory quality of food can’t be ignored (24). The safety and quality of cooked food could be improved by applying non-thermal technology conditioning prior to cooking. The novel heating technology for foods substitutes the conventional heating processes for providing thermal energy in cooking in addition to reducing cooking time, enriching quality factors, refining processing efficiency, and product safety. The prominent non-thermal processing techniques are highlighted by high hydrostatic pressure, UV-light, ultrasound, and pulsed electric fields that prove to be beneficial in ensuring the organoleptic as well as the nutritional constitution of food (23) (Figure 1).

It has been known for a long that processing at higher temperatures led to protein denaturation along with diminishing the water-holding capacity of meat (25) while structural deformations can be produced even at lower temperatures (26). When cooked at 58–64°C temperatures, the helical/crystalline structure of collagen molecules in meat shifts to a randomly coiled/amorphous one (27) which is accompanied by the bursting of hydrogen bonds resulting in the lowering of interaction between water and protein, relaxing of the structure of slender fiber along with tightening of the collagen structure. Unrestrained collagen fibers undergo shrinkage when subjected to a temperature treatment of 60–70°C (12), denaturation proceeding toward granulation, and increased solubilization followed by gelatinization due to the breakage of intermolecular bonds owing to an increase in heat (28, 29).

The high perishable rate of meat makes it highly imperative for its conservation by the following one or more processing techniques. In order to achieve this, various cooking methods used traditionally such as cooking in water and oven were put into practice since ancient times. Since these traditional methods have a few drawbacks such as a change in temperature between two locations within the meat, longer processing time, and reduced heating rate apart from a reduction in the overall quality of treated products. Amid this, various modernistic techniques have visualized evolution in recent years (30, 31). Commonly employed cooking methods such as boiling, roasting and grilling are accompanied by two methods of heat transfer. Heat transferred through the air in roasting or through water in sous-vide in a water bath involves a transfer by convection mode whereas in grilling meat is in direct contact with the heat source by conduction, which involves a comparatively shorter period of time for the cooking of meat (32).

With the increasing demand for high-quality foods, the application of non-thermal technologies has taken a spike in the food industry. The ultrasound treatments, high hydrostatic pressure processing, gamma and irradiation treatment are the most significantly implemented advanced industrial applications that have been potentially useful in inhibiting disease-producing microbes prevalent in meat and fish products (33). The aim of this review to measure the changes that occur in protein, sensory, chemical, and oxidative properties of meat during thermal and non-thermal processing techniques.

## 2. Thermal processing technologies

### 2.1. Roasting and grilling

Meat can be roasted by rotating it on a spit over a fire. Fat or oil is used as a basis because it is a dry heat technique. Direct grilling often exposes food to temperatures over 260°C. The Maillard reaction plays a critical role in the processing and cooking of foods, resulting in many chemical changes and being responsible for the generation of final colors and flavors (34). In a significant study, effects of grilling (230°C for 20 min) and roasting (190°C for 25 min) were analyzed on oxidation products of cholesterol in mutton and chicken using techniques such as saponification, extraction, derivatization followed by quantification by chromatographic analysis. Roasting treatment proved out to be much better as compared to grilling with respect to oxidation products of cholesterol. No significant difference was observed between raw mutton and raw chicken with respect to cholesterol with the former being more dominant than raw owing to high content of saturated fatty acids and cholesterol (35). Compared to other processing techniques, roasting increases lipid oxidation because it employs high temperatures for longer periods. Grilling, however, seems to have less impact on lipid oxidation than other cooking techniques (36).

This study aimed to elucidate the effects of cooking conditions on the physicochemical and sensory characteristics of dry- and wet-aged beef strip loins. Methods: Dry- and wet-aged beef aged for 28 days were cooked using different cooking methods (grilling or oven roasting) × cooking temperatures (150°C or 230°C), and their pH, 2-thiobarbituric acid reactive substances (TBARS), volatile compounds, and color were measured. Results: Cooking conditions did not affect pH; however, grilling resulted in lower TBARS but higher cooking doneness at the dry-aged beef surface compared to oven roasting. In descriptive sensory analysis, the roasted flavor of dry-aged beef was significantly stronger when grill-cooked compared to oven roasting. Dry-aged beef grill-cooked at 150°C presented a higher intensity of cheesy flavor, and that grilled at 230°C showed a greater intensity of roasted flavor compared to wet-aged beef at the same condition, respectively. Conclusion: Grilling may be effective for enhancing the unique flavor in dry-aged beef. In conclusion, the advantages of dry aging can be enhanced by grill cooking instead of oven roasting, as grilling improves desirable flavor and color. In addition, the grill-cooked dry aged beef might be appealing to consumers due to its intense roasted flavor, compared to grill-cooked wet-aged beef at the same cooking condition, and it is greater when cooking temperature is higher. Within the treatments in this study, grill cooking of dry-aged beef at a higher temperature (230°C) would be recommended (37).

Raw broiler charqui was subjecting to grilling, roasting, frying, and sous-vide techniques with sous-vide cooked samples exhibiting the lowest moisture loss than roasted and fried samples. High cooking temperature had significant effects on oxidation of proteins, tryptophan fluorescence, protein carbonylation, and disulfide bond formation of chicken charqui. Duration of cooking had a deep impact on free thiol groups, schiff base formation and hardness. Color of broiler charqui got affected adversely by the type of cooking technique which was attributed to Maillard reaction. In terms of quality, sous-vide technique proved out to be the most advantageous (38).

Chang et al. (39) assessed the impact of grilling and roasting on the concentration of seven polycyclic aromatic hydrocarbons, including chrysene, benzo(b)fluoranthene, benzo(k)fluoranthene, benzo(a)pyrene, dibenzo(a,h)anthracene, benzo(g,h,i)perylene, and indeno(1,2,3-c,d)pyrene in beef and pork meat, revealing that both grilling and roasting in beef and pig-induced polycyclic aromatic hydrocarbons. Roasting was evaluated to report the highest values of shear force for any type of meat measured by the Warner Blatzer test. While beef that had been boiled in a water bath displayed softness with shear force values that fell between roasted and grilled beef samples, meat that had been grilled and roasted showed substantial variations in tenderness.

It is important to understand the physical changes of meat texture during cooking, in fact, duration and temperature applied for cooking meat have a large effect on the physical properties of meat and eating quality (40). Moreover, tenderness is an important quality trait which determines satisfaction, repeat purchase and willingness-to-pay premium prices (41). Cooking is the final step prior to consumption and has a significant effect on sensory qualities (42). Heat-induced denaturation of major meat proteins, and actin and myosin denaturation have been associated with tougher meat, and collagen denaturation has been linked to a decrease in firmness (43). Heating temperature and rate also affect the extent of collagen denaturation, reporting that the temperature at which collagen denatured increased with the increased rate (44). Changes in these proteins are continuous with increasing temperature and, on the other hand, the level of tenderness fluctuates along the course of cooking, thus, protein conformational change alone cannot fully explain the tenderness of cooked meat (45).

### 2.2. Deep-frying

Being predominantly rich in water, lipids, proteins, vital amino acids, and several micronutrients including prominent minerals such as iron, magnesium, selenium, and zinc, meat is a crucial component of our diets. The majority of people take proteins as a key component of their diet (46). Deep-frying is a method of cooking that dates to centuries and has become widely patronized because of the uniqueness of its color and taste characteristics, becoming one of the most used cooking techniques also for meat (47). Deep-frying involves submerging food in heated oil or fat that has reached a high temperature, above the boiling point of water.

Different frying processes and their effects were studied on the nutritional, physicochemical and sensory profile of mackerel. Deep frying had significant adverse effects on lipid oxidation and protein degradation whereas overall acceptance score was incredibly increased by air frying. A reduction in perfluorinated compounds, few toxins, activity of trypsin inhibitor was exhibited during frying process apart from increase in the sensory parameters of mackerel. Lower rate of oxidation and retention of nutritional and sensory qualities of fried mackerel and minced mackerel products were reported in vacuum frying. An increase in volatile basic nitrogen and thiobarbituric acid reactive substances in mackerel was observed during frying. A huge impact was reported in the proximate composition as well as the mineral content of fried mackerel. The fatty acid profile of fried mackerel was also affected by the absorption of linoleic acid from oil, oxidative decomposition, and water loss. Amino acids were also decomposed by high temperature used in frying. This pivotal study concluded that frying process invariably influenced taste, color, and overall acceptance of mackerel (48).

Jin et al. (49) analyzed deep fat frying and hot air frying on texture, color difference, sensory score, and volatile flavor compounds of giant salamander meatballs prior to and after frying. Increased hardness, elasticity, and L* (brightness) whereas lower a*, b* value and fat content was reported in hot air-fried giant salamander meatballs. The relative content of aldehydes and ketones of fried giant salamander meatballs increased to a high extent. Sobowale et al. (50) investigated the effects of optimized cooking time (30, 45, 60 min), frying temperature (150, 170, 190°C) and time (3, 6, 9 min) on the moisture content, moisture loss, fat and protein content, color, textural, and sensory characteristics of deep fat fried goat meat sausage by using response surface methodology. The overall quality, texture, and sensory characteristics of goat meat sausage were significantly affected by the frying conditions. Nevertheless, the storage and microbiological studies of deep fat fried goat meat sausages need to be explored to a high extent. In comparison to other procedures, high temperature (frying) results in a greater loss of moisture. One of the most often used thermal techniques for chicken meatballs is deep frying. Additionally, various frying temperatures and periods were employed as research factors. The amount of heterocyclic aromatic amines created by this frying method is less than 1 ng/g. According to research, deeper frying at an elevated temperature for a prolonged period of time caused chicken meatballs to lose weight more quickly (46). The final protein digestibility of these foods depends on the ingredients added (i.e., meatballs contain flour), but however, some studies reported that the protein retention in meat after the deep-frying process ranged from 96 to 100% (47).

### 2.3. Steam cooking

The duration of steam cooking may vary from short duration of time (30–40 min) for small cuts of meat to longer durations for a whole leg of lamb (51). Notwithstanding the fact that the application of superheated steam in drying of food can render various avenues for research, there is still less reporting about its utilization in the preparation of quality meat products (52). Steam cooking is less time consuming and involves uniform distribution of heat (53). Quite similar to other processing techniques, steam cooking induces alterations in meat proteins *via* denaturation and protein aggregation affecting the digestibility of muscle proteins (54).

Rakotondramavo et al. (55) conducted steam cooking on cooked ham prepared from pork Longissimus dorsi up to core temperature of 65°C. The *in vitro* digestibility and the digestion rate after steam cooking was evaluated. The overall digestibility and rate of digestion of the meat proteins was enhanced in comparison to raw meat due to denaturation leading to exposure of the interior of the previously native molecules. In another significant study exhibiting the effect of superheated steam along with marination and smoking on meat, superheated steam treatment showed higher overall acceptance in quality in leg meat as well as breast meat viz. a viz. nutritional composition and wholesomeness during storage period despite microbial and chemical spoilage (56).

Abdulhameed et al. (57) applied kinetic modeling using superheated steam cooking to analyze the texture and color changes of chicken sausage for a time period of few minutes. A gradual reduction in texture was observed with increase in cooking time and the L* value (brightness or lightness) of meat decreased in a linear fashion with cooking time thus depicting a first-order kinetic model for texture parameters and L*-value of color for meat.

### 2.4. Pan-frying

A Teflon-coated pan with little oil or fat as the foundation is used for pan-frying to quickly cook meat. The pan should have adequate width to prevent the meat from becoming crowded while cooking. Overcrowding a small skillet with meat lowers the temperature and delays cooking. According to Liao et al. (58), the beef is cooked using this approach at 180°C for 5–10 min. As opposed to the other cooking procedures, boiling and pan-frying produced rabbit meat samples with considerably greater protein and fat contents and much lower moisture content. Differently, other authors reported that pork meat could be pan-fried with a temperature that ranges from 150 to 250°C, with great sensory profile consequences. It seems that by increasing the frying temperature, the intensity of the sensory fried and burnt attributes increased as the boiled and piggy notes decreased, reporting that at a lower temperature the aroma profile is correlated to lipid oxidation products, while ah higher ones it is correlated to Maillard reaction products (59). The amount of heterocyclic aromatic amines produced as a result of this method of frying ranges from 2 to 70 ng/g of cooked meat (28). When compared to other cooking procedures, samples undergoing boiling and pan-frying showed considerably higher total volatile base nitrogen values. The increased protein content achieved after thermal processing methods as compared to other cooking techniques may be the cause of the higher total volatile base nitrogen values found.

### 2.5. Sous-vide cooking

Although consumer taste for meat kinds, such as beef, hog and chicken, differs among nations, regions and people, the intake of meat derived from poultry has been continuously rising over the world since antiquity (60). The process known as sous-vide involves heating raw beef that has been vacuum-sealed in a water bath to a predetermined temperature (61). The method is often referred to as the “cook-in-bag” methodology. Sous-vide cooking uses temperatures typically between 50 and 85°C, which means that it needs more time to heat up than traditional cooking techniques. It is considered a novel way of cooking characterized by meat sealed in bag (often vacuum) and cooked in water bath with the aim to retain more moisture, flavor and natural state (62, 63), with no Maillard reaction products formation (54), conserving some superior technological characteristics such as oxidative stability (63). Moreover, the vacuum packaging is able to prevent losses of volatile compounds and moisture during cooking, giving a better sensory quality (64). It lessens the temperature gradient and lessens harm to vitamins and proteins that are sensitive to heat (65). Additionally, it minimizes cooking loss and maintains juiciness. Hardness and shear force values are dependent on cooking time and the relationship of time and temperature, chewiness and cohesiveness of meat are significantly affected by cooking time, temperature, and the interaction between the two factors (66). Some authors reported that the shear force declined when the cooking temperature increased from 50 to 65°C (61). In comparison to steam-cooked beef, sous-vide beef had a reduced percentage of polyunsaturated fatty acids, including n-3, but a similar n-6/n-3 ratio. In comparison to the control group, sous-vide samples subjected to cooking at 60°C (SV60) reported a decrease in pH value. Many modifications occurs to proteins during cooking (cross-linking, aggregation, oxidation, and changes in conformation) (32).

### 2.6. Microwave heating

It is a prominent thermal treatment encompassing low consumption of energy and shorter cooking time (67). It facilitates the sustainability and efficiency of industrial production process apart from preserving the nutritional profile of the product (68). Several physicochemical modifications in proteins occurring in thermal processing get manifested in the form of several quality parameters predominantly palatability, bioavailability, digestibility, and cooking efficiency (67). As compared to conventional techniques, innovative and advanced methods such as microwave and ohmic heating have proven to be causing lower denaturation of meat proteins at high heating rates (69). Microwave cooking converts electromagnetic energy into thermal energy and is employed to process several meat products such as bacon, meat balls, and patties (70). Microwave cooking induced an increase in textural hardness apart from increasing water holding capacity leading to a higher deterioration of myofibrillar and sarcoplasmic proteins (67).

Li et al. (71) reported significant levels of protein denaturation, rupturing of cell membranes, solubilization of connective tissue, and presence of large gaps between meat fibers and muscle bundles in yak meat. Less structural damage of muscles was found in meat samples treated with microwave cooking which was attributable attributed to the less duration of cooking time. Few disadvantages are also encountered during microwave cooking with respect to alterations in the quality attributes of the meat such as cooking defects and unacceptable structural properties (72). Dong et al. (73) reported the significant effect of microwave treatment on the secondary structures, *in vitro* protein digestibility and microstructural characteristics of shrimp proteins and concluded that the total soluble protein content and *in vitro* protein digestibility got significantly after undergoing microwave processing.

### 2.7. Ohmic heating

Several ohmic heating techniques that employ electrical current to heat food ingredients have been developed and patented for more than a century. However, the processing of foods with flowable liquid-particulates is the only commercial use. Ohmic cooking produced beef products that cooked more quickly used less energy, and were safer; ohmic heating generates heat volumetrically inside the material and can raise the temperature at a higher rate (74). This cooking method results in a firmer product, characterized by higher hardness and lower springier than that of the conventional method (75). Ohmic heating significantly decreased cooking loss and enhanced juiciness (17, 76, 77). Numerous studies have demonstrated that ohmic heating, either alone or in combination with traditional cooking techniques, may be utilized as a cooking process to produce beef products that are safer. In comparison to steam cooking, ohmically cooked beef exhibited a more invariably lighter and diminished red hue, a decrease in cooking loss, but a rougher texture. The content and physical characteristics of the food subjected to heating have a significant impact on the efficiency of ohmic heating. The formulation of meat products intended for ohmic heating requires an understanding of both meat and non-meat constituents due to their possible impact on ohmic heating. The inherent amounts of electrically conductive elements in raw meats are adequate to permit ohmic heating, but the composition of the additional additives can significantly change these levels. A small-scale ohmic heating cell was used to assess the values of various beef slices during ohmic heating (78). According to Shirsat et al. (79), adding lean to fat enhanced the conductivity overall. The heating of meat emulsions and meat batters has been accomplished using ohmic heating. While traditional beef had a cooking loss of 27%, ohmic low-temperature long-time-treated meat had the lowest cook loss (25.20%) (76). According to Zhang et al. (80), the shorter heating times may have reduced coagulating tendency of myofibrils and the gelating capacity of collagen, which would have reduced the ability of the ohmic heated samples for an efficient binding and entrapment of water within the cooked meat, explaining the lower water holding capacity of the ohmically cooked samples.

## 3. Non-thermal processing technologies

Nowadays, non-thermal processing techniques are being used for the purpose of food preservation. The most widely used non-thermal processing techniques are shown in Table 1.

**TABLE 1:** Summary of the application of various thermal and non-thermal processing techniques on various types of meat.

Treatment types	Types of meat involved	Key findings	References
Microwave processing	Shrimp proteins	Decrease in protein digestibility	(73)
	Beef filet	Absence of carcinogenic polyaromatic hydrocarbons in the samples subjected to steam and microwave preheating	(135)
	Rainbow trout protein	Increase in protein digestibility	(136)
	Chicken, beef, pork, kangaroo, trout, salmon, prawn, and oyster	Negligible effects on the digestibility of meat	(137)
	Chicken meat	Increase in water holding capacity (WHC) whereas a decrease in sarcoplasmic protein solubility was reported in thigh meat	(67)
Grilling	Corn, trout, beef, prawns, and pork	Polycyclic aromatic hydrocarbons (PAHs) emitted from thermal cooking were able to reach the vulnerable AL region of the lungs	(138)
	Beef satay	Fluoranthene concentration of total PAHs was found to be highest (132 ng/g) in beef satay	(139)
	Chicken proteins	Increase in protein digestibility	(140)
	Beef and salmon	Production of the high level of heterocyclic aromatic amines (HAs) and polycyclic aromatic hydrocarbons (PAHs) in grilled salmon sample	(141)
	Grilled beef, pork, chicken, turkey	Decrease in proteolytic action of pepsin during gastric digestion	(142)
	Whole muscles of Goose breast meat	Highest retention of minerals viz. Ca, Mg, Fe, Cu, and Mn	(8)
Stewing	Pork	Decrease in protein digestibility and large particle size	(143)
	Pork	Decrease in protein digestibility of stewed pork than the cooked pork	(144)
	Chicken proteins	Decrease in digestibility of muscle proteins after 3 h of stewing	(145)
	Beef proteins	Partial digestion of small peptides was reported after 30 min of stewing	(146)
Infra-red processing	Lean beef meatball, Turkey	Total polyaromatic hydrocarbons (PAHs) detected were below the prescribed EC limits	(147)
	Pork, Turkey Breast, and Corned Beef	High levels of aspartic acid, threonine, and serine levels	(148)
	Hamburger patties	Samples heated with far infra-red radiation suffer less cookout losses, giving a more desirable yield	(149)
	Biltong (dried and cured meat)	Enhancement of microbial quality and satisfactory tenderness	(150)
Sous-vide cooking	Beef	An increase in protein solubility and protein digestibility was reported	(151)
	Pork	Increase in protein digestibility	(143)
	Beef steaks	A decrease in the small molecular weight peptides was observed	(152)
	Beef proteins	Negligible effect on protein digestibility	(153)
Boiling	Beef frankfurters	Decrease in pH of meat during digestion	(154)
	Hairtail filets	Increase in protein digestibility
	Beef	Increase in digestion by proteases (trypsin and pepsin)	(155)
	Pork	Increase in digestibility of actomyosin	(156)
	Beef	Enhanced digestibility of muscle proteins	(157)
	Beef stripes	The concentration of benzofluoranthene, benzopyrene, indenopyrene, and benzoperylene was reported as 0.01–5.11 μg/kg	(158)
Pan frying	Beef	The high content of heterocyclic aromatic amines in pan-fried meat	(159)
	Minced beef	The addition of carbohydrates reduced the amount of heterocyclic amines	(16)
	Longissimus dorsi of beef	Control pan-fried samples reported a better odor and higher overall quality than the beer- and wine-marinated pan-fried steaks	(160)
	Hairtail fish proteins	Increase in protein digestibility	(161)
	Rabbit proteins	Decrease in protein digestibility	(162)
Ultrasound	Beef	Aggregation of myosin	(163)
	Squid mantle	Increase in hydrophobicity	(164)
	Chicken myofibrils	Negligible modification in primary structure of proteins	(165)
	Protein isolate from duck liver	Negligible modification in primary structure of proteins	(166)
High pressure processing	Tropomyosin from shrimp	Increase in surface hydrophobicity with increase in pressure	(167)
	Myofibrillar proteins from pork	Dynamic structural changes in soluble proteins, actin, and myosin due to aggregation	(168)
	Bovine *longissimus dorsi* muscle meat	Significant changes in the visual appearance and texture, higher breakdown of the parent proteins with high pressure (600 MPa), significant changes in myofibrillar structure	(169)
	Breast meats from Chicken	Conversion of α-helix and β-sheet into random coil and β-turn.	(27)
	*Trichiurus Haumela Surimi*	Conversion of α-helix into a random coil	(170)

### 3.1. Irradiation

High-energy gamma rays (60 Co and 137 Cs), X-rays, and accelerated electrons are the three ionizing radiation types that can be employed to irradiate food (81). The inactivation of microbes in food is more strongly impacted by changing the intensity of irradiation. Meat is also preserved using irradiation for a number of days. Inactivation of *L. monocytogenes*, *E. coli*, and *S. typhimurium* in ready-to-cook chicken under a storage period of 15 days when exposed to gamma radiation at intensities of 0, 1.5, 3, and 4.5 kGy produced outstanding results (82). Even after 15 days of storage, the chicken that was ready to eat still had pleasing sensory and textural qualities.

In addition to its potential role in extending shelf life by preventing food-borne illnesses and spoiling, irradiation technology is a desirable sterilizing technique due to consumer readiness to pay for processing for food safety. However, the use of irradiation, at higher doses, has been found to cause some unfavorable changes in food, especially in foods like meat, whose color and lipid content serves as the primary defining characteristics. Even a small change in color or lipid content may cause consumers to reject the product (83).

Irradiation alters food components, particularly the lipids and proteins in meat, although it can also preserve the original quality of meat (84). Fats begin to oxidize on their own when exposed to radiation, which produces rancid off-flavors. With increasing irradiation dose, the thiobarbituric acid reactive substances levels of pork patties packaged aerobically increased (85). Food irradiation doesn’t present any unique nutritional issues. Irradiation dosages up to 10 kGy do not appreciably impact the calorific value of macronutrients treated with radiations in food (86).

The consumer assumption that food irradiation involves nuclear technology restricts the growth of irradiation technology applications in the food industry sector. For the advancement of this technology, it is crucial to change customer perceptions and persuade them to purchase irradiated food, as well as to construct safer and more reliable equipment and optimize treatments (80).

### 3.2. Ultrasound

Recently, meat and fish have benefited from the use of ultrasound. Because it doesn’t generate waste and causes less harm to the environment and human health (87, 88), it is regarded as a green preservation technique. This process does not require chemical additives that can leach out into the water apart from less production of sewage (89).

Ultrasound is acoustic energy, moreover, it is a non-ionizing, non-invasive, and non-polluting form of mechanical energy (90), characterized by a great potential to control, improve, and accelerate processes without damaging the quality of food (87, 91).

Microorganisms may be rendered inactive by the ultrasound process, which can also alter the conformational structure of proteins, unfold the structure, and even splits the protein peptide chains through a process known as cavitation. This process produces cavitation bubbles that expand over several cycles before abruptly collapsing. This causes the cavitation zone to experience severe temperatures (over 5,000 K), high pressures (1,000 atm), high shear energy waves, free radical generation, and turbulence. Microorganisms are rendered inactive by the breakdown of microbial cells, while meat is rendered more tender by the breakdown of animal cells (92, 93). Furthermore, the cell damage improves the textural qualities without affecting the nutritional quality (94–96). The impact of ultrasound processing on actomyosin particle size was observed and was related to the protein dissociation during cavitation, which also resulted in the removal of aggregates and agglomerates.

High-intensity ultrasound processing has the power to disrupt cellular and subcellular components, and the cyclic oscillation of the acoustic pressure weakens cell membranes. Proteins, minerals and other components migrate because of tissue damage, which also accelerates enzyme activity. After receiving ultrasonic therapy, broiler breast muscle shear force values may be significantly reduced (86) by eliminating spoilage bacteria and improving product stability while in storage, the advantage of using ultrasound is that it extends shelf life. According to observations, the degradation of important quality indicators such as color and total phenolics, flavonoids, ascorbic acid and total antioxidant capacity as assessed by 2,2-diphenyl-1-picrylhydrazyl free radical scavenging activity is not significantly impacted by ultrasound processing (97). But ultrasound can also have detrimental impacts on food quality, such as the development of off-odors and the deterioration of substances associated with nutritional values.

### 3.3. Pulsating electric field

Using pulsating electric field technology, food can be preserved while also being shielded from the growth of pathogenic organisms. It allows for the inactivation of bacteria while preserving the natural color, taste and texture of uncooked food, as opposed to thermal treatment. The pulsating electric field method allows for the safe and effective increase of meat and meat products’ safety. Since the process happens swiftly and the pulse duration ranges from micro to milliseconds, the entire inactivation of microorganisms does not result in the heating of the conserved product (98). While pulsating electric field’s non-thermal nature guarantees the preservative parameter of high-quality meat and significant nutritional retention even at high temperatures, high temperatures, which are typically used as one of the principal food preservation methods, are to blame for the degradation of vitamins and bioactive components.

Given that the pulsating electric field works by exerting its effects on cellular changes and enhanced permeability of the cellular structure derived from electroporation, it is worthwhile to acknowledge its effect on significant nutrients viz. nutritional minerals such as iron, zinc, potassium and phosphorus, which have been reported for beef (99). The alteration of cellular structure may result in mineral loss either due to purging loss over time due to diffusion and lack of a physical obstruction due to pressure brought on by protein denaturation (100).

### 3.4. High-pressure processing

High-pressure processing is a non-thermal technology with several uses in the processing of meat, including tenderization and salt reduction. It is a method used to reduce the quality issues with low-fat meat products, and, more recently, is an innovative approach to increase digestibility. High-pressure processing also referred to as cold pasteurization of food pertains to that non-thermal treatment that is currently one of the most popular replacements for thermal food preservation techniques employed by the food industry. It is frequently used to treat meat and meat products, as the meat industry uses about 29% of all industrial high-pressure equipment (101).

High-pressure processing allows the extension of the shelf life along with preserving the raw materials’ intrinsic nutritive values and organoleptic qualities (such as taste, smell, color, and texture) (102). Muscle protein denaturation is induced by high-pressure processing therapy, which also alters protein unfolding and refolding, changing the protein’s structure and properties. When proteins are subjected to high-pressure processing, their denaturation patterns are unpredictable and depend on the amount of pressure used as well as the protein’s source. As a result, pressure-induced changes in protein structure can alter how easily proteins are absorbed during gastrointestinal digestion. Studies have shown that high-pressure processing facilitates the protein digestibility of a variety of foods, including muscle foods. Fresh meat’s usual characteristics, such as texture and notably color, can be significantly altered by high-pressure processing since it alters fresh meat’s quality criteria (103). According to reports, high-pressure processing treatment causes electrostatic connections to break down and stimulates sulfhydryl-disulfide bond exchange processes, which causes protein dissociation and unfolding of its structure. Intermolecular and intramolecular interactions lead to refolding following the treatment, which preserves the protein structure. Muscle proteins exhibit physiochemical changes like denaturation, dissociation, solubilization, aggregation, and gelation when subjected to high pressures, which modify their characteristics. Pressure, temperature, pH, and ionic strength all have a significant impact on these variables (104). According to studies, high-pressure processing causes significantly alters the color of fresh meat, although alterations in cured meat products are tolerable and rely on the product’s water content and water activity value (103). Although the processes underlying high-pressure processing-induced lipid oxidation is not completely understood, it has been proposed that high-pressure processing can facilitate lipid oxidation by making iron from hemoproteins more accessible and by causing membrane disruption (103). One of the most significant elements in the non-microbial deterioration of meat is oxidation (105). Lipid oxidation typically does not become apparent right away after high-pressure processing, but it can be noticed during chilled storage (106).

## 4. Changes in meat and meat products during various thermal and non-thermal processing techniques

**FIGURE 2:**
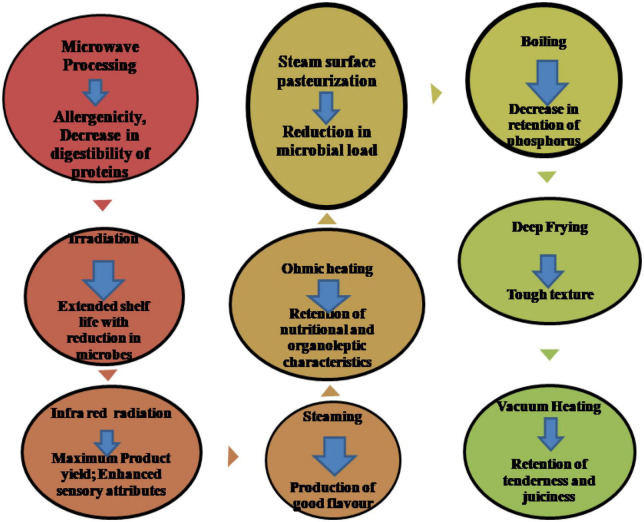
Various changes occur during meat processing.

Cooking temperature and duration impact the physical characteristics and eating quality of meat. The distinctive meat proteins are denatured during cooking, which is what causes the meat’s textural profile to undergo several structural alterations. Cell membranes are destroyed, meat fibers shrink, myofibrillar and sarcoplasmic proteins aggregate and form gels, and connective tissue shrinks and solubilizes because of these processes. Thermal treatment can lead to unfavorable changes in meat quality, including nutritive value loss due to lipid oxidation and modifications in a few protein segments (Table 2 and Figure 2).

**TABLE 2:** Changes during various thermal processing techniques in various types of meat.

Thermal processing techniques	Meat types	Processing condition (Time/temperature)	Changes during processing	References
Steaming	Sturgeon fish	37–100 for 4–20 min	The best profound flavor precursor was found at 12 min	(154)
Vacuum heating	Sturgeon fish	70°C for 30 min, 60°C for 15–30 min	Tenderness/juiciness	(164)
Steaming, boiling and air frying	Tilapia fish	Steaming (100°C for 5 min); Boiling (100°C for 5 min); Frying (200°C for 10 min)	Enhanced metabolites specifically arginine detected in the frying	(171)
Boiling, frying, and grilling	Bacon, chicken nuggets, tuna, chicken breast, beef	Boiling (90°C for 10 min); Grilling (150°C for 4–8 min); Frying (70°C for 10 min)	Highest N^ε^ -carboxymethyl-lysine (CML) (21.2 mg/kg) found in grilled tuna. Lowest N^ε^ -carboxymethyl-lysine (CML) (9.42 mg/kg) found in poached chicken	(172)
Boiling	Grass carp and catfish	100°C for 5, 10, 30 min	Negligible effects on the free advanced glycation end-products (AGEs) in fish muscle. Formation of protein-bound AGEs	(173)
Sterilization in an oil bath	Beef, pork, and chicken meat	121°C for 10 min	Insignificant effect on advanced glycation end-products (AGEs) in meat. 0.6- to 3.6 times increase in protein-bound AGEs	(174)
Sterilization and pasteurization	Pork meat	Sterilization (121°C for 30 min); Pasteurization (85°C for 4 h)	N^ε^ -carboxyethyl-lysine (CEL) generation detected in sterilization; N^ε^ -carboxymethyl-lysine (CML) formation in pasteurization	(30)
Microwave heating	Bovine supraspinatus muscle	900 W for 2.5–10 min	Increase in the darkening of meat samples	(175)
Ohmic heating	Pork meatball	Heating rate = 4.9°C/min, internal temperature (74°C); 72 V	Firmer uniform microstructure with bright colors	(176)
Infrared and microwave heating before freezing	Pork	Infrared (40°C, wavelength of 4–14 μm, power of 600 W for 90, 120, and 150 s.); Microwave (50, 100, and 150 W for 90 s)	Significant improvement in the quality of meat	(177)

Cooking techniques alter the physical and biochemical properties of the protein, carbohydrates, lipids, and other minor components of meat, which in turn affect yield, tenderness, juiciness, flavor, and palatability (107). The effects of five different cooking methods (boiling, steaming, grilling, microwaving, and superheated steaming) on proximate composition, pH, color, cooking loss, textural properties, and sensory characteristics of chicken steak were studied. Moisture content and lightness value (L*-value) were higher in superheated steam cooked chicken steak in comparison to chicken steaks subjected to boiling, steaming, grilling, and microwaving cooking. Superheated steam samples exhibited better sensory characteristics, tenderness, juiciness, and overall acceptability score. Thus, the application of superheated steam in a preheated 250°C oven and 380°C steam for 5 min ensured better quality characteristics and sensory properties of chicken steak (52).

The formation of another protein network and the thermal modifications taking place in muscle proteins during heating directly affect the yield, texture, and overall quality of the final product. Meat that is thermally tender after cooking results in toughening based on the cooking conditions (108). The level of shrinkage is a pivotal factor for the consumers preference as various thermal processing techniques alter the meat’s structure unfavorably, and more shrinkage is viewed as a sign of inferior quality (109). Low temperatures and longer time lead to an increase in shrinkage during thermal processing. The level of shrinkage is well augmented to increase with temperature, thereby leading to a huge water loss during cooking (12).

Amid these detrimental effects of thermal processing techniques on products, a wide array of non-thermal processing treatments for the inhibition of microbes have been developed to combat the global demand for nutritive and natural food items. Amongst non-thermal technologies, ultrasound, high-pressure processing, pulsed electric fields and pulsed light treatment stand out in immobilizing microorganisms at ambient temperatures. This preserves the food components from thermal degradation apart from maintaining the organoleptic and nutritional profile of the fresh-like food products (110).

Non-thermal processing encompasses all those processing techniques leading to the destruction of microorganisms along with preserving food from various harmful thermal effects and prolonging product shelf life by keeping intact their physical, nutritional, and sensory attributes. The inactivation of microorganisms can be achieved to variable degrees by high the application of processing technologies prominently hydrostatic pressure, pulsed electric fields, high-intensity ultrasound, ultraviolet light, pulsed light, ionizing radiation, and oscillating magnetic fields (111). Non-thermal processing is indeed one of those significant technologies which have been applied in the food sector for prolonging shelf life and preserving food integrity at reduced processing costs. Non-thermal technologies clearly assist the environment, whether by increasing the process’ total energy effectiveness or utilizing fewer non-renewable resources.

### 4.1. Changes in sensory attributes

Traditional cooking methods employ the application of high temperatures contributing to the destruction of nutritional components, flavor and color, etc. to a considerable extent (112). The distinguished aroma and flavor characteristics of meat during cooking are acquired due to the complex interaction of precursors originating from the lean and fat components of meat resulting in the development of volatile flavor compounds imparting a typical meat flavor (113). The sous-vide method is one of those methods which provides higher microbiological safety in comparison to conventional cooking (114). It leads to enhancement of tenderness, higher retention of color, and flavor, and reduction in destruction of protein, lipid, and heat-sensitive compounds (51). Ohmic heating for ground beef samples under different voltage gradients does not significantly affect the color of the samples. Such beef samples reflect more homogeneity in color and are devoid of a cooked crust layer on their surface (77).

The color of cooked meat is considered as a major determinant of safety and wholesomeness. An unacceptable brown color on the inside is an indicator of properly cooked meat whereas a pink appearance is correlated with uncooked ones (115). Color of meat is one of the significant parameters which is an indicator of the meat quality reflecting consumer’s preference can give reliable information about eating quality. Due to high temperature employed in cooking, an increase in hue angle values of cooked samples was reported as compared to raw samples. A kinetic model was developed to describe the texture and color changes of chicken sausage subjected to superheated steam cooking at temperature ranging from 150 to 200°C for 2–6 min. Hardness, cohesiveness, gumminess, and chewiness apart from brightness (L*), a* (redness) were evaluated. A gradual reduction in texture and brightness (L*) was reported with increase in cooking times and temperatures (57).

### 4.2. Chemical changes induced during processing

The myofibrils comprise of a mesh of protein networks of several different proteins in mutual interaction with each other thereby leading to stable protein complexes and muscle structures. The denaturation of globular heads of the myosin molecule starts at 40°C with structural modifications visible in myosin sub fragments alongside complete denaturation taking place above 53°C (116). Myosin starts the denaturation process simultaneously with the changes observed with low field NMR accompanied by a reduction in water-protein interactions leading to shrinkage and increased cook loss during the preliminary stages of low-temperature long-time cooking (117, 118). During heating at temperatures (58–64°C), there is a sharp transition in the collagen molecule from the crystalline (helical) state to an amorphous (randomly coiled) (119) which is mainly attributed to the breakage of hydrogen bonds resulting in the reduction of water-protein interactions followed by loosening of the fibrillar structure and contraction of the collagen molecule. Thermal treatment at temperatures of 60–70°C led to shrinkage of unrestrained collagen fibers (12). The denaturation process paves the way for granulation and increased solubilization and gelatinization owing to the breakage of intermolecular bonds with the application of intense heat (120, 121). During frying, it is the simultaneous heat and mass transfer of oil and air which induces a number of chemical changes viz. loss of moisture, formation of crust, uptake of oil, starch gelatinization, protein denaturation along with color changes induced by Maillard reactions and oil polymerization. Cooked meat acquires a characteristic texture by heat-induced changes in connective tissue, myofibrillar proteins and soluble proteins. The solubility of collagen molecules is dependent on the cross-linkage between collagen molecules within the connective tissue. These alterations in collagen solubility during thermal processing have a remarkable effect on the texture of poultry meat.

#### 4.2.1. Oxidative changes

**FIGURE 3:**
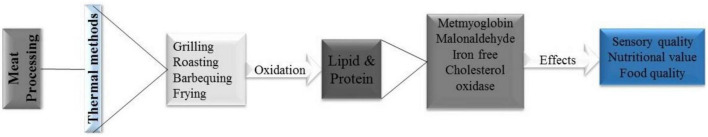
Oxidative changes after thermal processing.

The great water activity of meat and fish products is a great breeding ground for the growth and activity of spoilage and pathogenic microorganisms (122, 123). Due to this, a high level of reduction of essential nutritive elements of these matrices such as proteins, amino acids and essential vitamins predominantly vitamin B complex takes place along with lipid degradation which is susceptible toward oxidative degradative phenomena (124, 125). These are the free radicals which are responsible for the acceleration of lipid oxidation as well as protein denaturation (90, 126). Lipid oxidation in meat during cooking imparts a characteristic taste and odor to the meat apart from inducing product deterioration, production of undesirable odors, rancidity, nutritional losses, and toxin production (127, 128). The typical aroma and flavor of meats as a result of cooking originates from volatile flavor components produced by thermally induced reactions during heating *via* four pathways such as the Maillard reaction involving the combination of amino acid or peptides with reducing sugars; lipid oxidation; interaction between Maillard reaction products with lipid-oxidized products and deterioration of vitamins (129). Thermal processing is one of the pivotal factors which results in moisture loss in meat and is quite substantial from a sensory point of view with a high moisture loss being an indicator of less juiciness of meat (130). Oxidative changes after thermal processing as shown in Figure 3.

### 4.3. Changes in meat protein during thermal or non-thermal processing

**FIGURE 4:**
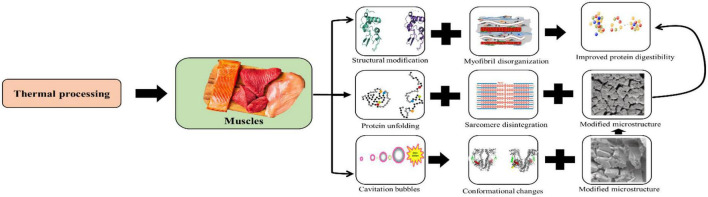
Effect on protein structure after thermal processing diagrammatically.

Manifold changes in the conformation in protein structure along with flavor, texture, and appearance, and the chemical properties take place during processing (Table 3). There are four different structures of proteins which have been identified viz. primary, secondary, tertiary, and quaternary. One of the most significant conformational changes prevalent in proteins upon the application of thermal treatment is the denaturation phenomena. During the heating process, the tertiary structure gets ruptured owing to external stress, predominately heat. Thermal processing also leads to the unfolding of the protein, rupturing of protein-protein association and finally leading to the destruction of collagen (12). Effect on protein structure after thermal processing as shown in Figure 4.

**TABLE 3:** Effect of various thermal processing methods on protein derived from various meats.

Meat types	Thermal processing	Processing conditions (Temperature/Time)	Changes during processing	References
Shrimp (*Litopenaeus vannamei*)	Microwave processing	75–125°C for 5–15 min	Decrease in allergenicity and digestibility of proteins. Increase in antioxidant capacity	(73)
N-labeled bovine meat	Cooking in a steam oven	90°C for 30 min; 55°C for 5 min	Low digestibility	(178)
Frankfurters (beef, pork, and turkey)	Steam surface pasteurization	1140°C for 1.5 s	Retention of minerals, and vitamins. Reduction in microbial count on the meat surface	(179)
Lamb meat	Roasting, frying, pan-frying, or stewing	Roasting (200°C for 5–45 min); Frying (200°C for 2, 3.5, 5, 6.5, and 8 min); Pan frying (200°C for 2.0–8.0 min); Stewing (100°C for 1–6 h)	Higher heterocyclic aromatic amine content was detected in stewing	(180)
Lamb	Deep frying	180°C for 1–1.5 min	Desirable color and tough texture	(181)
Lamb	Smoking	Cooking (85°C for 30 min); Smoking (50°C for 20 min)	Reduction in heterocyclic aromatic amine	(182)
Frankfurter (beef + pork)	Smoking	Smoking (52°C for 10 min), Drying (56°C for 12 min)	Highest polycyclic aromatic hydrocarbons contents during smoking	(183)
Beef	Dry air and steam	The temperature of core (75, 85, 95°C)	Desirable tenderness and juiciness	(184)
Fresh beef and chicken breast	Boiling	10, 20, and 30 min	Greater nutritional peptides derivation and less retention of phosphorous	(185)
Pork	Grilling	200–260°C	Formation of heterocyclic aromatic amines and polycyclic aromatic hydrocarbons, retention of sensory properties	(186)
Skinless deboned chicken breast meat	Infra-red radiation and superheated steam	130–170°C	Maximum product yield, positive sensory attributes at 170°C	(187)
Minced beef	Irradiation	1–4 kGy	Extended shelf life with the reduction in microbial growth	(86)
Beef, pork, lamb, chicken, and turkey	Ohmic heating	3.5 kW, 15 A, 0–240 V, 50 Hz	Negligible structural, nutritional, or organoleptic alterations	(76)
Chicken patties	Steam-assisted hybrid oven	180–240°C	Reduction in the amount of carcinogenic compounds	(188)
Marinated lamb loins	Sous-vide cooking	60°C for 12 h	Minimized water loss and a firmer texture	(189)

While heating between 40 and 60°C extending up to 90°C, aggregation of most sarcoplasmic proteins takes place. These heat-induced aggregated sarcoplasmic proteins can lead to the formation of gel within the constituents of the structure of meat resulting in the development of consistency in meat after cooking (131). Gel formation takes place with the denaturation of myofibrillar proteins in solution at a low concentration. This process commences with the rupturing of hydrogen bonds resulting in the opening up of the fibrillar structure followed by the contraction of the collagen molecule. When meat is heated at 60–80°C for 1-h, significant changes in the structure of proteins took place. Collagen in the epimysium remained unaffected after heating whereas the perimysial and the endomysial collagen attain granular structure at 60°C while the gelatinization process started at 80°C (46). A significant deviation was seen in the solubilization of collagen during the heating process. Thermal processing techniques such as cooking, roasting, grilling, frying, boiling, and sterilization have proven their potential in destroying pathogens but are accompanied by deteriorative effects on food composition and characteristics. The degree of denaturation in proteins during heating is dependent on the temperature and the protein under consideration resulting in the destruction of their quaternary and tertiary structures apart from the development of unfolded random shapes (132).

Different non-thermal processing technologies such as irradiation, ultrasound, cold plasma, pulsed electric field, and high-pressure treatments also tend to induce changes in the protein structure, solubility, and other functional properties (116). The primary structure of proteins is indicated by the amount and type of amino acids present in it. The formation of cross-links by disulfide bonds is the mechanism responsible for myosin aggregation. The ultrasound treatment has led to the cavitation phenomena formed due to free radicals by disulfide bonds (133). Few other findings have reported the significance of covalent and non-covalent bonds as the main interactions leading to the aggregation of muscle proteins subjected to ultrasound treatment. High levels of structural modifications were induced due to the destruction of bonds between amino acids and some distinct parts of proteins succeeded by protein aggregation followed by the degradation of inter-and intra-molecular hydrogen bonds in β-sheet and α-helix structures, respectively. This led to unfolding apart from the transition of a random structure into random coils and β-turns (134).

## 5. Conclusion

Textural properties of meat are an indication of several factors predominantly- animal, environmental, and managemental and processing ones. The protein structure of the meat is modified by phenomena such as denaturation, Maillard reaction or aggregation. Thermal processing techniques inclusive of commercial and domestic, modify the muscle proteins resulting in alterations in digestibility. Nevertheless, thermal processing of the meat ensures microbial safety to a considerable extent, it hampers the digestibility and bioavailability of nutrients during gastrointestinal digestion. Proper selection of operating conditions has noticable consequences not only on the meat quality but also on the efficiency of the cooking process. Improvement of the current cooking practices or investigating new cooking strategies is essential for the meat processing industry. Notwithstanding the fact that the traditional cooking techniques help in improving the appearance and taste of the lamb meat, these traditional methods such as grilling, smoking, roasting, etc. also pose several constraints in ensuring consumer health. The evaluation of the optimum cooking process for ensuring high quality and energy-efficient meat cooking aid the consumer to select an efficient cooking method and processing parameter of meat cooking. Inhibition of lipid oxidation in meat and meat products is quite imperative to the food industry for retaining the sensory and nutritional profile of meat and meat products. Novel processing techniques primarily microwave cooking involves the reduction in the energy requirement leading to less cooking losses. Apart from microwave cooking, it is infrared grilling and ohmic heating which can help to mitigate toxic compounds thus improving the sensory quality and ensuring the microbial safety of meat. Non-thermal treatments such as microencapsulation, high-pressure processing, and pulsed electric fields, irradiation have now become the most focused areas of research in the food sector in light of consumer cravings for nutritious, wholesome, and microbial-free food.

## Author contributions

WK, JK, and MSA: conceptualization. MFA, NA, and MSA: methodology. PM and WK: software. WK, JK, SK, MSA, AM, and PD: validation, investigation, and data curation. WK, JK, SK, MSA, and PD: formal analysis. WK, JK, SK, K-u-WZ, MSA, AM, and PD: resources. AM, WK, K-u-WZ, SK, and JK: writing—original draft preparation. WK, SK, AM, and PD: writing—review and editing and supervision. WK, JK, SK, AM, and PD: visualization. WK, SK, and PD: project administration and funding acquisition. All authors have read and agreed to the published version of the manuscript.

## References

[1] Khalid W Arshad MS Yasin M Imran A Ahmad MH. Quality characteristics of gamma irradiation and kale leaf powder treated ostrich and chicken meat during storage. *Int J Food Properties.* (2021) 24:1335–48. 10.1080/10942912.2021.1963274

[2] Jayathilakan K Sultana K Radhakrishna K Bawa AS. Utilization of byproducts and waste materials from meat, poultry and fish processing industries: a review. *J Food Sci Technol.* (2012) 49:278–93. 10.1007/s13197-011-0290-7 23729848 PMC3614052

[3] De Palo P Maggiolino A Centoducati P Tateo A. Slaughtering age effect on carcass traits and meat quality of italian heavy draught horse foals. *Asian-Australas J Anim Sci.* (2013) 26:1637–43. 10.5713/ajas.2013.13174 25049752 PMC4093806

[4] De Palo P Maggiolino A Centoducati P Milella P Calzaretti G Tateo A. Is meat quality from Longissimus lumborum samples correlated with other cuts in horse meat? *Anim Prod Sci.* (2016) 87:428–38. 10.1111/asj.12433 26464235

[5] De Palo P Tateo A Maggiolino A Marino R Ceci E Nisi A Martina Franca donkey meat quality: influence of slaughter age and suckling technique. *Meat Sci.* (2017) 134:128–34. 10.1016/j.meatsci.2017.07.025 28783609

[6] De Palo P Maggiolino A Centoducati P Calzaretti G Ceci E Tateo A. An assessment of sire-breed effects on carcass and meat quality traits of lambs at the ages of 40 and 100 days from Comisana ewes crossed with Suffolk or Bergamasca rams. *J Anim Prod Sci.* (2018) 58:1794–801. 10.1071/AN16673

[7] Gálvez F Maggiolino A Domínguez R Pateiro M Gil S De Palo P Nutritional and meat quality characteristics of seven primal cuts from 9-month-old female veal calves: a preliminary study. *J Sci Food Agric.* (2019) 99:2947–56. 10.1002/jsfa.9508 30471118

[8] Maggiolino A Pateiro M Serrano MP Landete-Castillejos T Domínguez R García A Carcass and meat quality characteristics from Iberian wild red deer (*Cervus elaphus*) hunted at different ages. *J Sci Food Agric.* (2019) 99:1938–45. 10.1002/jsfa.9391 30270485

[9] Gálvez F Domínguez R Maggiolino A Pateiro M Carballo J De Palo P Meat quality of commercial chickens reared in different production systems: industrial, range and organic. *Ann Anim Sci.* (2020) 20:263–85. 10.1093/femsle/fnz092 31123750

[10] Maggiolino A Bragaglio A Salzano A Rufrano D Claps S Sepe L Dietary supplementation of suckling lambs with anthocyanins: effects on growth, carcass, oxidative and meat quality traits. *Anim Feed Sci Technol.* (2021) 276:114925. 10.1016/j.anifeedsci.2021.114925

[11] Maggiolino A Sgarro MF Natrella G Lorenzo J Colucci A Faccia M Dry-aged beef steaks: effect of dietary supplementation with pinus taeda hydrolyzed lignin on sensory profile. *Colorimetr Oxid Stabil.* (2021) 10:1080. 10.3390/foods10051080 34068173 PMC8152972

[12] Tornberg E. Effects of heat on meat proteins – Implications on structure and quality of meat products. *Meat Sci.* (2005) 70:493–508. 10.1016/j.meatsci.2004.11.021 22063748

[13] Alfaia CMM Alves SP Lopes AF Fernandes MJE Costa ASH Fontes CMGA Effect of cooking methods on fatty acids, conjugated isomers of linoleic acid and nutritional quality of beef intramuscular fat. *Meat Sci.* (2010) 84:769–77. 10.1016/j.meatsci.2009.11.014 20374855

[14] de Jonge R. Predictable and unpredictable survival of foodborne pathogens during non-isothermal heating. *Int J Food Microbiol.* (2019) 291:151–60. 10.1016/j.ijfoodmicro.2018.11.018 30502585

[15] Roccato A Uyttendaele M Cibin V Barrucci F Cappa V Zavagnin P Survival of *Salmonella* Typhimurium in poultry-based meat preparations during grilling, frying and baking. *Int J Food Microbiol.* (2015) 197:1–8. 10.1016/j.ijfoodmicro.2014.12.007 25540842

[16] Raza A Shabbir MA Khan MI Suleria HAR Sultan S. Effect of thermal treatments on the formation of heterocyclic aromatic amines in various meats. *J. Food Process. Preserv.* (2015) 39:376–83. 10.1111/jfpp.12242

[17] Özkan N Ho I Farid M. Combined ohmic and plate heating of hamburger patties: quality of cooked patties. *J Food Eng.* (2004) 63:141–5. 10.1016/S0260-8774(03)00292-9

[18] Ježek F Kameník J Macharáčková B Bogdanovičová K Bednář J. Cooking of meat: effect on texture, cooking loss and microbiological quality – a review. *Acta Veterinaria Brno.* (2019) 88:487–96. 10.2754/avb201988040487 22026406

[19] Tateo A Maggiolino A Domínguez R Lorenzo JM Dinardo FR Ceci E Volatile organic compounds, oxidative and sensory patterns of vacuum aged foal meat. *Animals.* (2020) 10:1495. 10.3390/ani10091495 32847084 PMC7552191

[20] Sgarro MF Maggiolino A Pateiro M Domínguez R Iannaccone F De Palo P Effects of anthocyanin supplementation and ageing time on the volatile organic compounds and sensory attributes of meat from goat kids. *Animals.* (2022) 12:139. 10.3390/ani12020139 35049761 PMC8772539

[21] Singh L Agarwal T Simal-Gandara J. PAHs, diet and cancer prevention: cooking process driven-strategies. *Trends Food Sci Technol.* (2020) 99:487–506. 10.1016/j.tifs.2020.03.030

[22] Goluch Z Barbara K Haraf G Wołoszyn J Okruszek A Wereńska M. Impact of various types of heat processing on the energy and nutritional values of goose breast meat. *Poultry Sci.* (2021) 100:101473. 10.1016/j.psj.2021.101473 34607154 PMC8496166

[23] Chen F Zhang M Fan K Mujumdar AS. Non-thermal technology and heating technology for fresh food cooking in the central kitchen processing: a review. *Food Rev Int.* (2022) 38:608–27. 10.1080/87559129.2020.1740246

[24] Lagnika C Zhang M Mothibe KJ. Effects of ultrasound and high pressure argon on physico-chemical properties of white mushrooms (*Agaricus bisporus*) during postharvest storage. *Postharvest Biol Technol.* (2013) 82:87–94. 10.1016/j.postharvbio.2013.03.006

[25] Naqvi ZB Thomson PC Ha M Campbell MA McGill DM Friend MA Effect of sous vide cooking and ageing on tenderness and water-holding capacity of low-value beef muscles from young and older animals. *Meat Sci.* (2021) 175:108435. 10.1016/j.meatsci.2021.108435 33461157

[26] Sun X Li X Tang J Lai K Rasco BA Huang Y. Formation of protein-bound Nε-carboxymethyllysine and Nε-carboxyethyllysine in ground pork during commercial sterilization as affected by the type and concentration of sugars. *Food Chem.* (2021) 336:127706. 10.1016/j.foodchem.2020.127706 32768907

[27] Dominguez-Hernandez E Salaseviciene A Ertbjerg P. Low-temperature long-time cooking of meat: eating quality and underlying mechanisms. *Meat Sci.* (2018) 143:104–13. 10.1016/j.meatsci.2018.04.032 29730528

[28] Abdel-Naeem HHS Sallam KI Zaki HMBA. Effect of different cooking methods of rabbit meat on topographical changes, physicochemical characteristics, fatty acids profile, microbial quality and sensory attributes. *Meat Sci.* (2021) 181:108612. 10.1016/j.meatsci.2021.108612 34171787

[29] Argel NS Lorenzo G Domínguez R Fraqueza MJ Fernández-López J Sosa ME Hybrid meat products: incorporation of white bean flour in lean pork burgers. *Appl Sci.* (2022) 12:7571. 10.3390/app12157571

[30] Yu L Gao C Zeng M He Z Wang L Zhang S Effects of raw meat and process procedure on Nε-carboxymethyllysine and Nε-carboxyethyl-lysine formation in meat products. *Food Sci Biotechnol.* (2016) 25:1163–8. 10.1007/s10068-016-0185-5 30263389 PMC6049122

[31] Yu L Li Y Gao C Yang Y Zeng M Chen J. Nε-carboxymethyl-lysine and Nε-carboxyethyl-lysine contents in commercial meat products. *Food Res Int.* (2022) 155:111048. 10.1016/j.foodres.2022.111048 35400433

[32] Yu T-Y Morton JD Clerens S Dyer JM. Cooking-induced protein modifications in meat. *Compr Rev Food Sci Food Saf.* (2017) 16:141–59. 10.1111/1541-4337.12243 33371543

[33] Al-Nehlawi A Guri S Guamis B Saldo J. Synergistic effect of carbon dioxide atmospheres and high hydrostatic pressure to reduce spoilage bacteria on poultry sausages. *LWT Food Sci Technol.* (2014) 58:404–11. 10.1016/j.lwt.2014.03.041

[34] Arihara K Yokoyama I Ohata M. Bioactivities generated from meat proteins by enzymatic hydrolysis and the Maillard reaction. *Meat Sci.* (2021) 180:108561. 10.1016/j.meatsci.2021.108561 34034035

[35] Alina A Ah NM Shazamawati Z Nurulhuda M Hs US Imtinan A. Effect of grilling and roasting on the fatty acids profile of chicken and mutton. *World Appl Sci J.* (2012) 17:29–33.

[36] Dominguez R Gomez M Fonseca S Lorenzo JM. Effect of different cooking methods on lipid oxidation and formation of volatile compounds in foal meat. *Meat Sci.* (2014) 97:223–30. 10.1016/j.meatsci.2014.01.023 24583332

[37] Lee D Lee HJ Yoon JW Ryu M Jo C. Effects of cooking conditions on the physicochemical and sensory characteristics of dry- and wet-aged beef. *Anim Biosci.* (2021) 34:1705–16. 10.5713/ab.20.0852 33561325 PMC8495346

[38] Silva FAP Ferreira VCS Madruga MS Estévez M. Effect of the cooking method (grilling, roasting, frying and sous-vide) on the oxidation of thiols, tryptophan, alkaline amino acids and protein cross-linking in jerky chicken. *J Food Sci Technol.* (2016) 53:3137–46. 10.1007/s13197-016-2287-8 27784908 PMC5055878

[39] Chang H Wang Q Xu X Li C Huang M Zhou G Effect of heat-induced changes of connective tissue and collagen on meat texture properties of beef semitendinosus muscle. *Int J Food Properties.* (2011) 14:381–96. 10.1080/10942910903207728

[40] Combes S Lepetit J Darche B Lebas F. Effect of cooking temperature and cooking time on Warner–Bratzler tenderness measurement and collagen content in rabbit meat. *Meat Sci.* (2004) 66:91–6. 10.1016/S0309-1740(03)00019-6 22063936

[41] Gál R Kameník J Salek RN Polášek Z Macharáčková B Valenta T Research note: impact of applied thermal treatment on textural, and sensory properties and cooking loss of selected chicken and turkey cuts as affected by cooking technique. *Poultry Sci.* (2022) 101:101923. 10.1016/j.psj.2022.101923 35679669 PMC9189220

[42] Warner RD Wheeler TL Ha M Li X Bekhit AE-D Morton J Meat tenderness: advances in biology, biochemistry, molecular mechanisms and new technologies. *Meat Sci.* (2022) 185:108657. 10.1016/j.meatsci.2021.108657 34998162

[43] Martens H Stabursvik E Martens M. Texture and colour changes ini meat during cooking related to thermal denaturation of muscle proteins. *J Texture Stud.* (1982) 13:291–309. 10.1111/j.1745-4603.1982.tb00885.x

[44] Latorre ME Palacio MI Velázquez DE Purslow PP. Specific effects on strength and heat stability of intramuscular connective tissue during long time low temperature cooking. *Meat Sci.* (2019) 153:109–16. 10.1016/j.meatsci.2019.03.016 30925447

[45] Vaskoska R Ha M Naqvi ZB White JD Warner RD. Muscle, ageing and temperature influence the changes in texture, cooking loss and shrinkage of cooked beef. *Foods.* (2020) 9:1289. 10.3390/foods9091289 32937816 PMC7555138

[46] Shabbir MA Raza A Anjum FM Khan MR Suleria HAR. Effect of thermal treatment on meat proteins with special reference to heterocyclic aromatic amines (HAAs). *Crit Rev Food Sci Nutr.* (2015) 55:82–93. 10.1080/10408398.2011.647122 24915407

[47] Ananey-Obiri D Matthews L Azahrani MH Ibrahim SA Galanakis CM Tahergorabi R. Application of protein-based edible coatings for fat uptake reduction in deep-fat fried foods with an emphasis on muscle food proteins. *Trends Food Sci Technol.* (2018) 80:167–74. 10.1016/j.tifs.2018.08.012

[48] Negara BFSP Lee M-J Tirtawijaya G Cho W-H Sohn J-H Kim J-S Application of deep, vacuum, and air frying methods to fry chub mackerel (*Scomber japonicus*). *Processes.* (2021) 9:1225. 10.3390/pr9071225

[49] Jin W Pei J Chen X Geng J Chen D Gao R. Influence of frying methods on quality characteristics and volatile flavor compounds of giant salamander (*Andrias davidianus*) meatballs. *J Food Qual.* (2021) 2021:8450072. 10.1155/2021/8450072

[50] Sobowale SS Olayanju TA Mulaba-Bafubiandi AF. Process optimization and kinetics of deep fat frying conditions of sausage processed from goat meat using response surface methodology. *Food Sci Nutr.* (2019) 7:3161–75. 10.1002/fsn3.1145 31660130 PMC6804765

[51] Suleman R Wang Z Aadil RM Hui T Hopkins DL Zhang D. Effect of cooking on the nutritive quality, sensory properties and safety of lamb meat: current challenges and future prospects. *Meat Sci.* (2020) 167:108172. 10.1016/j.meatsci.2020.108172 32422547

[52] Choi YS Hwang KE Jeong TJ Kim YB Jeon KH Kim EM Comparative study on the effects of boiling, steaming, grilling, microwaving and superheated steaming on quality characteristics of marinated chicken steak. *Korean J Food Sci Anim Resourc.* (2016) 36:1–7. 10.5851/kosfa.2016.36.1.1 27499656 PMC4973943

[53] Pathare PB Roskilly AP. Quality and energy evaluation in meat cooking. *Food Eng Rev.* (2016) 8:435–47. 10.1007/s12393-016-9143-5

[54] Bhat ZF Morton JD Bekhit AEA Kumar S Bhat HF. Thermal processing implications on the digestibility of meat, fish and seafood proteins. *Compr Rev Food Sci Food Saf.* (2021) 20:4511–48. 10.1111/1541-4337.12802 34350699

[55] Rakotondramavo A Rabesona H Brou C de Lamballerie M Pottier L. Ham processing: effects of tumbling, cooking and high pressure on proteins. *Eur Food Res Technol.* (2019) 245:273–84. 10.1007/s00217-018-3159-4

[56] Cho W-H Choi J-S. Sensory quality evaluation of superheated steam-treated chicken leg and breast meats with a combination of marination and hot smoking. *Foods.* (2021) 10:1924. 10.3390/foods10081924 34441701 PMC8392690

[57] Abdulhameed AA Yang TA Abdulkarim A. Kinetics of texture and colour changes in chicken sausage during superheated steam cooking. *Polish J Food Nutr Sci.* (2016) 66:199–209. 10.1515/pjfns-2015-0044

[58] Liao GZ Wang GY Xu XL Zhou GH. Effect of cooking methods on the formation of heterocyclic aromatic amines in chicken and duck breast. *Meat Sci.* (2010) 85:149–54. 10.1016/j.meatsci.2009.12.018 20374878

[59] Meinert L Andersen LT Bredie WLP Bjergegaard C Aaslyng MD. Chemical and sensory characterisation of pan-fried pork flavour: interactions between raw meat quality, ageing and frying temperature. *Meat Sci.* (2007) 75:229–42. 10.1016/j.meatsci.2006.07.004 22063654

[60] Zhang W Naveena BM Jo C Sakata R Zhou G Banerjee R Technological demands of meat processing–An Asian perspective. *Meat Sci.* (2017) 132:35–44. 10.1016/j.meatsci.2017.05.008 28648604

[61] Vaudagna SR Sánchez G Neira MS Insani EM Picallo AB Gallinger MM Sous vide cooked beef muscles: effects of low temperature–long time (LT–LT) treatments on their quality characteristics and storage stability. *Int J Food Sci Technol.* (2002) 37:425–41. 10.1046/j.1365-2621.2002.00581.x

[62] Sun S Sullivan G Stratton J Bower C Cavender G. Effect of HPP treatment on the safety and quality of beef steak intended for sous vide cooking. *LWT.* (2017) 86:185–92. 10.1016/j.lwt.2017.07.037

[63] Roascio - Albistur A Gámbaro A. Consumer perception of a non-traditional market on sous-vide dishes. *Int J Gastron Food Sci.* (2018) 11:20–4. 10.1016/j.ijgfs.2017.10.002

[64] Baldwin DE. Sous vide cooking: a review. *Int J Gastron Food Sci.* (2012) 1:15–30. 10.1016/j.ijgfs.2011.11.002

[65] Di Giorgio L Salgado PR Mauri AN. Flavored oven bags for cooking meat based on proteins. *LWT* (2019) 101:374–81. 10.1016/j.lwt.2018.11.002

[66] Botinestean C Hossain M Mullen AM Kerry JP Hamill RM. The influence of the interaction of sous-vide cooking time and papain concentration on tenderness and technological characteristics of meat products. *Meat Sci.* (2021) 177:108491. 10.1016/j.meatsci.2021.108491 33761399

[67] Taşkıran M Olum E Candoğan K. Changes in chicken meat proteins during microwave and electric oven cooking. *J Food Proc Preserv.* (2020) 44:e14324. 10.1111/jfpp.14324

[68] Chizoba Ekezie F-G Sun D-W Han Z Cheng J-H. Microwave-assisted food processing technologies for enhancing product quality and process efficiency: a review of recent developments. *Trends Food Sci Technol.* (2017) 67:58–69. 10.1016/j.tifs.2017.05.014

[69] Hassoun A Heia K Lindberg SK Nilsen H. Spectroscopic techniques for monitoring thermal treatments in fish and other seafood: a review of recent developments and applications. *Foods.* (2020) 9:767. 10.3390/foods9060767 32532043 PMC7353598

[70] Orsat V Raghavan GSV Krishnaswamy K. 5 - Microwave technology for food processing: an overview of current and future applications. Second ed. In: Regier M Knoerzer K Schubert H editors. *The Microwave Processing of Foods.* Sawston: Woodhead Publishing (2017). p. 100–16. 10.1016/B978-0-08-100528-6.00005-X

[71] Li S Tang S Yan L Li R. Effects of microwave heating on physicochemical properties, microstructure and volatile profiles of yak meat. *J Appl Anim Res.* (2019) 47:262–72. 10.1080/09712119.2019.1624553

[72] Barbosa-Cánovas GV Medina-Meza I Candoğan K Bermúdez-Aguirre D. Advanced retorting, microwave assisted thermal sterilization (MATS), and pressure assisted thermal sterilization (PATS) to process meat products. *Meat Sci.* (2014) 98:420–34. 10.1016/j.meatsci.2014.06.027 25060584

[73] Dong X Wang J Raghavan V. Impact of microwave processing on the secondary structure, in-vitro protein digestibility and allergenicity of shrimp (*Litopenaeus vannamei*) proteins. *Food Chem.* (2021) 337:127811. 10.1016/j.foodchem.2020.127811 32799155

[74] Gavahian M Tiwari BK Chu Y-H Ting Y Farahnaky A. Food texture as affected by ohmic heating: mechanisms involved, recent findings, benefits, and limitations. *Trends Food Sci Technol.* (2019) 86:328–39. 10.1016/j.tifs.2019.02.022

[75] Icier F Izzetoglu GT Bozkurt H Ober A. Effects of ohmic thawing on histological and textural properties of beef cuts. *J Food Eng.* (2010) 99:360–5. 10.1016/j.jfoodeng.2010.03.018

[76] Zell M Lyng JG Cronin DA Morgan DJ. Ohmic cooking of whole beef muscle – Optimisation of meat preparation. *Meat Sci.* (2009) 81:693–8. 10.1016/j.meatsci.2008.11.012 20416569

[77] Bozkurt H Icier F. Ohmic cooking of ground beef: effects on quality. *J Food Eng.* (2010) 96:481–90.

[78] Sarang S Sastry SK Knipe L. Electrical conductivity of fruits and meats during ohmic heating. *J Food Eng.* (2008) 87:351–6. 10.1016/j.jfoodeng.2007.12.012

[79] Shirsat N Lyng JG Brunton NP McKenna B. Ohmic processing: electrical conductivities of pork cuts. *Meat Sci.* (2004) 67:507–14. 10.1016/j.meatsci.2003.12.003 22061526

[80] Zhang Z-H Wang L-H Zeng X-A Han Z Brennan CS. Non-thermal technologies and its current and future application in the food industry: a review. *Int J Food Sci Technol.* (2019) 54:1–13. 10.1111/ijfs.13903

[81] Farkas J. Irradiation for better foods. *Trends Food Sci Technol.* (2006) 17:148–52. 10.1016/j.tifs.2005.12.003

[82] Ibrahim HM. Prediction of meat and meat products by gamma rays, electron beams and X-ray irradiations–A review. *Int J Agric Sci.* (2013) 3:521–34.

[83] Urbain W. *Food Irradiation.* Amsterdam: Elsevier (2012).

[84] Jia W Shi Q Shi L. Effect of irradiation treatment on the lipid composition and nutritional quality of goat meat. *Food Chem.* (2021) 351:129295. 10.1016/j.foodchem.2021.129295 33631611

[85] Ahn DU Jo C Du M Olson DG Nam KC. Quality characteristics of pork patties irradiated and stored in different packaging and storage conditions. *Meat Sci.* (2000) 56:203–9. 10.1016/s0309-1740(00)00044-9 22061910

[86] Formanek Z Lynch A Galvin K Farkas J Kerry JP. Combined effects of irradiation and the use of natural antioxidants on the shelf-life stability of overwrapped minced beef. *Meat Sci.* (2003) 63:433–40. 10.1016/s0309-1740(02)00063-3 22062512

[87] Awad TS Moharram HA Shaltout OE Asker D Youssef MM. Applications of ultrasound in analysis, processing and quality control of food: a review. *Food Res Int.* (2012) 48:410–27. 10.1016/j.foodres.2012.05.004

[88] Turantaş F Kılıç GB Kılıç B. Ultrasound in the meat industry: general applications and decontamination efficiency. *Int J Food Microbiol.* (2015) 198:59–69. 10.1016/j.ijfoodmicro.2014.12.026 25613122

[89] Arroyo C Eslami S Brunton NP Arimi JM Noci F Lyng JG. An assessment of the impact of pulsed electric fields processing factors on oxidation, color, texture, and sensory attributes of turkey breast meat. *Poult Sci.* (2015) 94:1088–95. 10.3382/ps/pev097 25810409

[90] Chemat F Zille H Khan MK. Applications of ultrasound in food technology: processing, preservation and extraction. *Ultrason Sonochem.* (2011) 18:813–35. 10.1016/j.ultsonch.2010.11.023 21216174

[91] Gallego-Juarez JA. High-power ultrasonic processing: recent developments and prospective advances. *Phys Proc.* (2010) 3:35–47. 10.1016/j.phpro.2010.01.006

[92] Jayasooriya SD Bhandari BR Torley P D’Arcy BR. Effect of high power ultrasound waves on properties of meat: a review. *Int J Food Properties.* (2004) 7:301–19. 25974043

[93] Alarcon-Rojo AD Janacua H Rodriguez JC Paniwnyk L Mason TJ. Power ultrasound in meat processing. *Meat Sci.* (2015) 107:86–93. 10.1016/j.meatsci.2015.04.015 25974043

[94] O’Donnell CP Tiwari BK Bourke P Cullen PJ. Effect of ultrasonic processing on food enzymes of industrial importance. *Trends Food Sci Technol.* (2010) 21:358–67. 10.1016/j.tifs.2010.04.007

[95] Multari S Stewart D Russell WR. Potential of fava bean as future protein supply to partially replace meat intake in the human diet. *Comprehens Rev Food Sci Food Saf.* (2015) 14:511–22. 10.1111/1541-4337.12146

[96] Rosario DKA Rodrigues BL Bernardes PC Conte-Junior CA. Principles and applications of non-thermal technologies and alternative chemical compounds in meat and fish. *Crit Rev Food Sci Nutr.* (2021) 61:1163–83. 10.1080/10408398.2020.1754755 32319303

[97] Aadil RM Zeng X-A Han Z Sun D-W. Effects of ultrasound treatments on quality of grapefruit juice. *Food Chem.* (2013) 141:3201–6. 10.1016/j.foodchem.2013.06.008 23871078

[98] Al-juhaimi F Ghafoor K Özcan MM Jahurul MHA Babiker EE Jinap S Effect of various food processing and handling methods on preservation of natural antioxidants in fruits and vegetables. *J Food Sci Technol.* (2018) 55:3872–80. 10.1007/s13197-018-3370-0 30228385 PMC6133851

[99] Bhat ZF Morton JD Mason SL Bekhit AE-DA. Pulsed electric field: role in protein digestion of beef Biceps femoris. *Innov Food Sci Emerg Technol.* (2018) 50:132–8. 10.1016/j.ifset.2018.09.006

[100] Khan AA Randhawa MA Carne A Mohamed Ahmed IA Barr D Reid M Effect of low and high pulsed electric field on the quality and nutritional minerals in cold boned beef M. longissimus et lumborum. *Innov Food Sci Emerg Technol.* (2017) 41:135–43. 10.1016/j.ifset.2017.03.002

[101] Aymerich T Picouet PA Monfort JM. Decontamination technologies for meat products. *Meat Sci.* (2008) 78:114–29. 10.1016/j.meatsci.2007.07.007 22062101

[102] Ribeiro JS Santos MJMC Silva LKR Pereira LCL Santos IA da Silva Lannes SC Natural antioxidants used in meat products: a brief review. *Meat Sci.* (2019) 148:181–8. 10.1016/j.meatsci.2018.10.016 30389412

[103] Bajovic B Bolumar T Heinz V. Quality considerations with high pressure processing of fresh and value added meat products. *Meat Sci.* (2012) 92:280–9. 10.1016/j.meatsci.2012.04.024 22608831

[104] Sazonova S Galoburda R Gramatina I. Application of high-pressure processing for safety and shelf-life quality of meat–a review. *Proceedings of the 11th Baltic Conference on Food Science and Technology “FOODBALT”.* Jelgava: (2017). 10.22616/FoodBalt.2017.001

[105] Cepero-Betancourt Y Opazo-Navarrete M Janssen AEM Tabilo-Munizaga G Pérez-Won M. Effects of high hydrostatic pressure (HHP) on protein structure and digestibility of red abalone (Haliotis rufescens) muscle. *Innov Food Sci Emerg Technol.* (2020) 60:102282. 10.1016/j.ifset.2019.102282

[106] Guyon C Meynier A de Lamballerie M. Protein and lipid oxidation in meat: a review with emphasis on high-pressure treatments. *Trends Food Sci Technol.* (2016) 50:131–43. 10.1016/j.tifs.2016.01.026

[107] Mir NA Rafiq A Kumar F Singh V Shukla V. Determinants of broiler chicken meat quality and factors affecting them: a review. *J Food Sci Technol.* (2017) 54:2997–3009. 28974784 10.1007/s13197-017-2789-z PMC5603000

[108] Li C Wang D Dong H Xu W Gao F Zhou G Effects of different cooking regimes on the microstructure and tenderness of duck breast muscle. *J Sci Food Agric.* (2013) 93:1979–85. 10.1002/jsfa.6003 23239107

[109] Barbera S Tassone S. Meat cooking shrinkage: measurement of a new meat quality parameter. *Meat Sci.* (2006) 73(3):467–74. 10.1016/j.meatsci.2006.01.011 22062485

[110] Pereira RN Vicente AA. Environmental impact of novel thermal and non-thermal technologies in food processing. *Food Res Int.* (2010) 43:1936–43. 10.1016/j.foodres.2009.09.013

[111] Sunil NC Singh J Chandra S Chaudhary V Kumar V. Non-thermal techniques: application in food industries” A review. *J Pharmacognosy Phytochem.* (2018) 7:1507–18.

[112] Iborra-Bernad C Philippon D García-Segovia P Martínez-Monzó J. Optimizing the texture and color of sous-vide and cook-vide green bean pods. *LWT Food Sci Technol.* (2013) 51:507–13. 10.1016/j.lwt.2012.12.001

[113] Van Ba H Hwang I Jeong D Touseef A. Principle of meat aroma flavors and future prospect. *J Latest Res Qual Control.* (2012) 2:145–76.

[114] Ayub H Ahmad A. Physiochemical changes in sous-vide and conventionally cooked meat. *Int J Gastron Food Sci.* (2019) 17:100145.

[115] Suman SP Nair MN Joseph P Hunt MC. Factors influencing internal color of cooked meats. *Meat Sci.* (2016) 120:133–44. 27131513 10.1016/j.meatsci.2016.04.006

[116] Liu J Ruusunen M Puolanne E Ertbjerg P. Effect of pre-rigor temperature incubation on sarcoplasmic protein solubility, calpain activity and meat properties in porcine muscle. *LWT Food Sci Technol.* (2014) 55:483–9.

[117] Bertram HC Aaslyng MD Andersen HJ. Elucidation of the relationship between cooking temperature, water distribution and sensory attributes of pork – a combined NMR and sensory study. *Meat Sci.* (2005) 70:75–81. 10.1016/j.meatsci.2004.12.002 22063282

[118] Christensen M Ertbjerg P Failla S Sañudo C Richardson RI Nute GR Relationship between collagen characteristics, lipid content and raw and cooked texture of meat from young bulls of fifteen European breeds. *Meat Sci.* (2011) 87:61–5. 10.1016/j.meatsci.2010.09.003 20870360

[119] Lepetit J. A theoretical approach of the relationships between collagen content, collagen cross-links and meat tenderness. *Meat Sci.* (2007) 76:147–59. 10.1016/j.meatsci.2006.10.027 22064201

[120] Palka K. The influence of post-mortem ageing and roasting on the microstructure, texture and collagen solubility of bovine semitendinosus muscle. *Meat Sci.* (2003) 64:191–8. 10.1016/s0309-1740(02)00179-1 22062866

[121] Voutila L Ruusunen M Puolanne E. Comparison of the thermal characteristics of connective tissue in loose structured and normal structured porcine M. semimembranosus. *Meat Sci.* (2008) 80:1024–30. 10.1016/j.meatsci.2008.04.021 22063832

[122] McAfee AJ McSorley EM Cuskelly GJ Moss BW Wallace JMW Bonham MP Red meat consumption: an overview of the risks and benefits. *Meat Sci.* (2010) 84:1–13. 20374748 10.1016/j.meatsci.2009.08.029

[123] Olmedilla-Alonso B Jiménez-Colmenero F Sánchez-Muniz FJ. Development and assessment of healthy properties of meat and meat products designed as functional foods. *Meat Sci.* (2013) 95:919–30. 23623320 10.1016/j.meatsci.2013.03.030

[124] Cao JJ Nielsen FH. Acid diet (high-meat protein) effects on calcium metabolism and bone health. *Curr Opin Clin Nutr Metab Care.* (2010) 13:698–702. 10.1097/MCO.0b013e32833df691 20717017

[125] Manthey-Karl M Lehmann I Ostermeyer U Rehbein H Schröder U. Meat composition and quality assessment of king scallops (*Pecten maximus*) and frozen atlantic sea scallops (*Placopecten magellanicus*) on a retail level. *Foods.* (2015) 4:524–46. 10.3390/foods4040524 28231221 PMC5224553

[126] Fallas-Padilla K Rodriguez-Rodriguez C Jaramillo HF Enchandi MLA. *Arcobacter*: comparison of isolation methods, diversity, and potential pathogenic factors in commercially retailed chicken breast meat from costa rica. *J Food Protect.* (2014) 77:880–4. 10.4315/0362-028X.JFP-13-368 24853508

[127] Broncano JM Petrón MJ Parra V Timón ML. Effect of different cooking methods on lipid oxidation and formation of free cholesterol oxidation products (COPs) in Latissimus dorsi muscle of Iberian pigs. *Meat Sci.* (2009) 83:431–7. 10.1016/j.meatsci.2009.06.021 20416691

[128] Hyeon J-Y Chon J-W Hwang I-G Kwak H-S Kim M-S Kim S-K Prevalence, antibiotic resistance, and molecular characterization of *Salmonella* serovars in retail meat products. *J Food Protect.* (2011) 74:161–6. 10.4315/0362-028X.JFP-10-327 21219782

[129] Khan MI Jo C Tariq MR. Meat flavor precursors and factors influencing flavor precursors—A systematic review. *Meat Sci.* (2015) 110:278–84. 10.1016/j.meatsci.2015.08.002 26319308

[130] Bertram HC Andersen HJ Karlsson AH Horn P Hedegaard J Nørgaard L Prediction of technological quality (cooking loss and Napole Yield) of pork based on fresh meat characteristics. *Meat Sci.* (2003) 65:707–12. 10.1016/S0309-1740(02)00272-3 22063431

[131] Chen Y-C Jaczynski J. Protein recovery from rainbow trout (Oncorhynchus mykiss) processing byproducts via isoelectric solubilization/precipitation and its gelation properties as affected by functional additives. *J Agric Food Chem.* (2007) 55:9079–88. 10.1021/jf071992w 17902629

[132] Esteghlal S Gahruie HH Niakousari M Barba FJ Bekhit AE-D Mallikarjunan K Bridging the knowledge gap for the impact of non-thermal processing on proteins and amino acids. *Foods.* (2019) 8:262. 10.3390/foods8070262 31319521 PMC6678513

[133] Kang Z-L Li B Ma H-J Chen F-S. Effect of different processing methods and salt content on the physicochemical and rheological properties of meat batters. *Int J Food Properties.* (2016) 19:1604–15.

[134] Zhou K Canning C Sun S. Effects of rice protein hydrolysates prepared by microbial proteases and ultrafiltration on free radicals and meat lipid oxidation. *LWT Food Sci Technol.* (2013) 50:331–5.

[135] Farhadian A Jinap S Hanifah HN Zaidul IS. Effects of meat preheating and wrapping on the levels of polycyclic aromatic hydrocarbons in charcoal-grilled meat. *Food Chem.* (2011) 124:141–6.

[136] Nguyen E Jones O Kim YHB San Martin-Gonzalez F Liceaga AM. Impact of microwave-assisted enzymatic hydrolysis on functional and antioxidant properties of rainbow trout Oncorhynchus mykiss by-products. *Fish Sci.* (2017) 83:317–31.

[137] Luo J Taylor C Nebl T Ng K Bennett LE. Effects of macro-nutrient, micro-nutrient composition and cooking conditions on in vitro digestibility of meat and aquatic dietary proteins. *Food Chem.* (2018) 254:292–301. 10.1016/j.foodchem.2018.01.164 29548456

[138] Saito E Tanaka N Miyazaki A Tsuzaki M. Concentration and particle size distribution of polycyclic aromatic hydrocarbons formed by thermal cooking. *Food Chem.* (2014) 153:285–91. 10.1016/j.foodchem.2013.12.055 24491732

[139] Farhadian A Jinap S Abas F Sakar ZI. Determination of polycyclic aromatic hydrocarbons in grilled meat. *Food Control.* (2010) 21:606–10.

[140] Ferreira VCS Morcuende D Madruga MS Silva FAP Estévez M. Role of protein oxidation in the nutritional loss and texture changes in ready-to-eat chicken patties. *Int J Food Sci Technol.* (2018) 53:1518–26.

[141] Viegas O Novo P Pinto E Pinho O Ferreira IM. Effect of charcoal types and grilling conditions on formation of heterocyclic aromatic amines (HAs) and polycyclic aromatic hydrocarbons (PAHs) in grilled muscle foods. *Food Chem Toxicol.* (2012) 50:2128–34. 10.1016/j.fct.2012.03.051 22459130

[142] Martini S Conte A Tagliazucchi D. Comparative peptidomic profile and bioactivities of cooked beef, pork, chicken and turkey meat after in vitro gastro-intestinal digestion. *J Proteom.* (2019) 208:103500. 10.1016/j.jprot.2019.103500 31454557

[143] Li L Liu Y Zou X He J Xu X Zhou G In vitro protein digestibility of pork products is affected by the method of processing. *Food Res Int.* (2017) 92:88–94. 28290301 10.1016/j.foodres.2016.12.024

[144] He J Zhou G Bai Y Wang C Zhu S Xu X The effect of meat processing methods on changes in disulfide bonding and alteration of protein structures: impact on protein digestion products. *RSC Adv.* (2018) 8:17595–605. 10.1039/c8ra02310g 35539241 PMC9080411

[145] Qi J Li X Zhang W Wang H Zhou G Xu X. Influence of stewing time on the texture, ultrastructure and in vitro digestibility of meat from the yellow-feathered chicken breed. *Anim Sci J.* (2018) 89:474–82. 10.1111/asj.12929 29082572

[146] Kaur L Maudens E Haisman DR Boland MJ Singh H. Microstructure and protein digestibility of beef: the effect of cooking conditions as used in stews and curries. *LWT Food Sci Technol.* (2014) 55:612–20.

[147] Kendirci P Icier F Kor G Onogur TA. Influence of infrared final cooking on polycyclic aromatic hydrocarbon formation in ohmically pre-cooked beef meatballs. *Meat Sci.* (2014) 97:123–9. 10.1016/j.meatsci.2014.01.020 24576771

[148] Unklesbay N Davis ME Krause G. Nutrient retention in pork, turkey breast and corned beef roasts after infrared and convective heat processing. *J Food Sci.* (1983) 48:865–8.

[149] Sheridan P Shilton N. Application of far infra-red radiation to cooking of meat products. *J Food Eng.* (1999) 41:203–8.

[150] Cherono K Mwithiga G Schmidt S. Infrared drying as a potential alternative to convective drying for biltong production. *Italian J Food Saf.* (2016) 5:5625. 10.4081/ijfs.2016.5625 27853706 PMC5090110

[151] Adeyeye SAO. Heterocyclic amines and polycyclic aromatic hydrocarbons in cooked meat products: a review. *Polycyclic Aromatic Compounds.* (2020) 40:1557–67.

[152] Baugreet S Gomez C Auty MAE Kerry JP Hamill RM Brodkorb A. In vitro digestion of protein-enriched restructured beef steaks with pea protein isolate, rice protein and lentil flour following sous vide processing. *Innov Food Sci Emerg Technol.* (2019) 54:152–61.

[153] Šimko P. Factors affecting elimination of polycyclic aromatic hydrocarbons from smoked meat foods and liquid smoke flavorings. *Mol Nutr Food Res.* (2005) 49:637–47. 15945119 10.1002/mnfr.200400091

[154] Li X Xie W Bai F Wang J Zhou X Gao R Influence of thermal processing on flavor and sensory profile of sturgeon meat. *Food Chem.* (2022) 374:131689. 10.1016/j.foodchem.2021.131689 34875433

[155] Semedo Tavares WP Dong S Yang Y Zeng M Zhao Y. Influence of cooking methods on protein modification and in vitro digestibility of hairtail (*Thichiurus lepturus*) fillets. *LWT.* (2018) 96:476–81.

[156] Sayd T Chambon C Santé-Lhoutellier V. Quantification of peptides released during in vitro digestion of cooked meat. *Food Chem.* (2016) 197:1311–23. 10.1016/j.foodchem.2015.11.020 26675873

[157] Zhao D He J Zou X Xie Y Xu X Zhou G Influence of hydrothermal treatment on the structural and digestive changes of actomyosin. *J Sci Food Agric.* (2019) 99:6209–18. 10.1002/jsfa.9893 31250450

[158] Farouk MM Wu G Frost DA Staincliffe M Knowles SO. Factors affecting the digestibility of beef and consequences for designing meat-centric meals. *J Food Qual.* (2019) 2019:2590182.

[159] Olatunji OS Fatoki OS Opeolu BO Ximba BJ. Determination of polycyclic aromatic hydrocarbons [PAHs] in processed meat products using gas chromatography – Flame ionization detector. *Food Chem.* (2014) 156:296–300. 10.1016/j.foodchem.2014.01.120 24629971

[160] Persson E Sjöholm I Nyman M Skog K. Addition of various carbohydrates to beef burgers affects the formation of heterocyclic amines during frying. *J Agric Food Chem.* (2004) 52:7561–6. 10.1021/jf0493831 15675804

[161] Ozvural EB Bornhorst GM. Chemical and structural characteristics of frankfurters during in vitro gastric digestion as influenced by cooking method and severity. *J Food Eng.* (2018) 229:102–8.

[162] Melo A Viegas O Petisca C Pinho O Ferreira IM. Effect of beer/red wine marinades on the formation of heterocyclic aromatic amines in pan-fried beef. *J Agric Food Chem.* (2008) 56:10625–32. 10.1021/jf801837s 18950185

[163] Zhang Y Wang X Wang W Zhang J. Effect of boiling and frying on nutritional value and in vitro digestibility of rabbit meat. *Afr J Food Sci.* (2014) 8:92–103. 10.1002/jsfa.11773 35038172

[164] Wei J Chen Y Dong X He F Shi Y Chai T. Water holding capacity and microstructure of sturgeon (*Acipenser gueldenstaedti*) fillets as affected by low temperature vacuum heating. *Int J Food Properties.* (2021) 24:1061–73.

[165] Higuera-Barraza OA Torres-Arreola W Ezquerra-Brauer JM Cinco-Moroyoqui FJ Rodríguez Figueroa JC Marquez-Ríos E. Effect of pulsed ultrasound on the physicochemical characteristics and emulsifying properties of squid (Dosidicus gigas) mantle proteins. *Ultrason Sonochem.* (2017) 38:829–34. 10.1016/j.ultsonch.2017.01.008 28109677

[166] Wang J-Y Yang Y-L Tang X-Z Ni W-X Zhou L. Effects of pulsed ultrasound on rheological and structural properties of chicken myofibrillar protein. *Ultrason Sonochem.* (2017) 38:225–33. 28633822 10.1016/j.ultsonch.2017.03.018

[167] Zou Y Wang L Li P Cai P Zhang M Sun Z Effects of ultrasound assisted extraction on the physiochemical, structural and functional characteristics of duck liver protein isolate. *Process Biochem.* (2017) 52:174–82.

[168] Jin Y Deng Y Qian B Zhang Y Liu Z Zhao Y. Allergenic response to squid (Todarodes pacificus) tropomyosin Tod p1 structure modifications induced by high hydrostatic pressure. *Food Chem Toxicol.* (2015) 76:86–93. 10.1016/j.fct.2014.12.002 25530105

[169] Grossi A Olsen K Bolumar T Rinnan Å Øgendal LH Orlien V. The effect of high pressure on the functional properties of pork myofibrillar proteins. *Food Chem.* (2016) 196:1005–15. 10.1016/j.foodchem.2015.10.062 26593583

[170] Kaur L Astruc T Vénien A Loison O Cui J Irastorza M High pressure processing of meat: effects on ultrastructure and protein digestibility. *Food Funct.* (2016) 7:2389–97. 10.1039/c5fo01496d 27143217

[171] Li R Sun Z Zhao Y Li L Yang X Cen J Application of UHPLC-Q-TOF-MS/MS metabolomics approach to investigate the taste and nutrition changes in tilapia fillets treated with different thermal processing methods. *Food Chem.* (2021) 356:129737. 10.1016/j.foodchem.2021.129737 33836358

[172] Gómez-Ojeda A Jaramillo-Ortíz S Wrobel K Wrobel K Barbosa-Sabanero G Luevano-Contreras C Comparative evaluation of three different ELISA assays and HPLC-ESI-ITMS/MS for the analysis of Nε-carboxymethyl lysine in food samples. *Food Chem.* (2018) 243:11–8. 10.1016/j.foodchem.2017.09.098 29146316

[173] Niu L Sun X Tang J Wang J Rasco BA Lai K Free and protein-bound Nε-carboxymethyllysine and Nε-carboxyethyllysine in fish muscle: biological variation and effects of heat treatment. *J Food Composit Anal.* (2017) 57:56–63.

[174] Sun X Tang J Wang J Rasco BA Lai K Huang Y. Formation of free and protein-bound carboxymethyllysine and carboxyethyllysine in meats during commercial sterilization. *Meat Sci.* (2016) 116:1–7. 10.1016/j.meatsci.2016.01.009 26829237

[175] Musto M Faraone D Cellini F Musto E. Changes of DNA quality and meat physicochemical properties in bovine supraspinatus muscle during microwave heating. *J Sci Food Agric.* (2014) 94:785–91. 10.1002/jsfa.6441 24122804

[176] Engchuan W Jittanit W Garnjanagoonchorn W. The ohmic heating of meat ball: modeling and quality determination. *Innov Food Sci Emerg Technol.* (2014) 23:121–30.

[177] Hu R Zhang M Mujumdar AS. Application of infrared and microwave heating prior to freezing of pork: effect on frozen meat quality. *Meat Sci.* (2022) 189:108811. 10.1016/j.meatsci.2022.108811 35398771

[178] Oberli M Marsset-Baglieri A Airinei G Santé-Lhoutellier V Khodorova N Rémond D High true ileal digestibility but not postprandial utilization of nitrogen from bovine meat protein in humans is moderately decreased by high-temperature, long-duration cooking. *J Nutr.* (2015) 145:2221–8. 26290008 10.3945/jn.115.216838

[179] Murphy RY Hanson RE Johnson NR Chappa K Berrang ME. Combining organic acid treatment with steam pasteurization to eliminate listeria monocytogenes on fully cooked frankfurters. *J Food Protect.* (2006) 69:47–52. 10.4315/0362-028x-69.1.47 16416900

[180] Guo H Wang Z Pan H Li X Chen L Rao W Effects of traditional chinese cooking methods on formation of heterocyclic aromatic amines in lamb patties. *Food Sci Biotechnol.* (2014) 23:747–53.

[181] Iqbal SZ Talib NH Hasnol NDS. Heterocyclic aromatic amines in deep fried lamb meat: the influence of spices marination and sensory quality. *J Food Sci Technol.* (2016) 53:1411–7. 10.1007/s13197-015-2137-0 27570265 PMC4984708

[182] Hou C Wang Z Wu L Chai J Song X Wang W Effects of breeds on the formation of heterocyclic aromatic amines in smoked lamb. *Int. J. Food Sci. Technol.* (2017) 52:2661–9.

[183] Pöhlmann M Hitzel A Schwägele F Speer K Jira W. Influence of different smoke generation methods on the contents of polycyclic aromatic hydrocarbons (PAH) and phenolic substances in Frankfurter-type sausages. *Food Control.* (2013) 34:347–55. 10.1016/j.meatsci.2011.06.024 21764224

[184] Modzelewska-Kapituła M Dąbrowska E Jankowska B Kwiatkowska A Cierach M. The effect of muscle, cooking method and final internal temperature on quality parameters of beef roast. *Meat Sci.* (2012) 91:195–202. 10.1016/j.meatsci.2012.01.021 22336137

[185] Cupisti A Comar F Benini O Lupetti S D’Alessandro C Barsotti G Effect of boiling on dietary phosphate and nitrogen intake. *J Renal Nutr.* (2006) 16:36–40. 10.1053/j.jrn.2005.10.005 16414439

[186] Pan H Cao Y. Optimization of pretreatment procedures for analysis of polycyclic aromatic hydrocarbons in charcoal-grilled pork. *Analyt. Lett.* (2009) 43:97–109.

[187] Suwannakam M Noomhorm A Anal AK. Influence of combined far-infrared and superheated steam for cooking chicken meat patties. *J Food Proc Preserv.* (2014) 37:515–23.

[188] Isleroglu H Kemerli T Özdestan Ö Üren A Kaymak-Ertekin F. Effect of oven cooking method on formation of heterocyclic amines and quality characteristics of chicken patties: steam-assisted hybrid oven versus convection ovens. *Poultry Sci.* (2014) 93:2296–303. 10.3382/ps.2013-03552 24974393

[189] Roldan M Loebner J Degen J Henle T Antequera T Ruiz-Carrascal J. Advanced glycation end products, physico-chemical and sensory characteristics of cooked lamb loins affected by cooking method and addition of flavour precursors. *Food Chem.* (2015) 168:487–95. 10.1016/j.foodchem.2014.07.100 25172739

